# Cardiac gene therapy treats diabetic cardiomyopathy and lowers blood glucose

**DOI:** 10.1172/jci.insight.166713

**Published:** 2023-09-22

**Authors:** Jing Li, Bradley Richmond, Ahmad A. Cluntun, Ryan Bia, Maureen A. Walsh, Kikuyo Shaw, J. David Symons, Sarah Franklin, Jared Rutter, Katsuhiko Funai, Robin M. Shaw, TingTing Hong

**Affiliations:** 1 Department of Pharmacology and Toxicology, College of Pharmacy,; 2 Nora Eccles Harrison Cardiovascular Research and Training Institute,; 3 Department of Biochemistry,; 4 College of Health, Department of Nutrition and Integrative Physiology, Program in Molecular Medicine,; 5 Diabetes & Metabolism Research Center, and; 6 Howard Hughes Medical Institute, University of Utah, Salt Lake City, Utah, USA.

**Keywords:** Cardiology, Cell Biology, Gene therapy, Glucose metabolism, Heart failure

## Abstract

Diabetic cardiomyopathy, an increasingly global epidemic and a major cause of heart failure with preserved ejection fraction (HFpEF), is associated with hyperglycemia, insulin resistance, and intracardiomyocyte calcium mishandling. Here we identify that, in db/db mice with type 2 diabetes–induced HFpEF, abnormal remodeling of cardiomyocyte transverse-tubule microdomains occurs with downregulation of the membrane scaffolding protein cardiac bridging integrator 1 (cBIN1). Transduction of cBIN1 by AAV9 gene therapy can restore transverse-tubule microdomains to normalize intracellular distribution of calcium-handling proteins and, surprisingly, glucose transporter 4 (GLUT4). Cardiac proteomics revealed that AAV9-cBIN1 normalized components of calcium handling and GLUT4 translocation machineries. Functional studies further identified that AAV9-cBIN1 normalized insulin-dependent glucose uptake in diabetic cardiomyocytes. Phenotypically, AAV9-cBIN1 rescued cardiac lusitropy, improved exercise intolerance, and ameliorated hyperglycemia in diabetic mice. Restoration of transverse-tubule microdomains can improve cardiac function in the setting of diabetic cardiomyopathy and can also improve systemic glycemic control.

## Graphical abstract

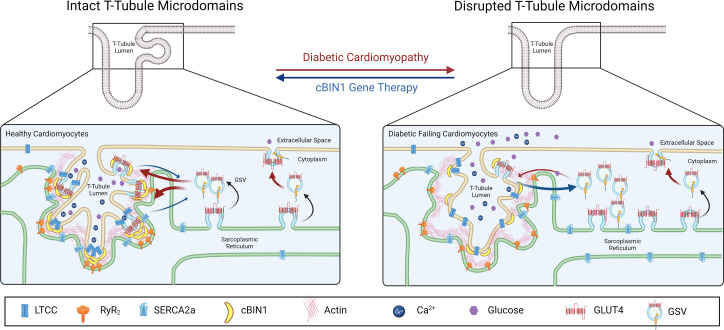

## Introduction

There are currently 451 million people diagnosed with diabetes worldwide, 90% of whom have type 2 diabetes mellitus (T2DM) with heart failure as the most common cardiovascular complication (1). Affecting over one-third of patients with diabetes, diabetic cardiomyopathy is a growing cause of heart failure and cardiac premature mortality (1). Furthermore, most patients with diabetic cardiomyopathy have symptoms that manifest as heart failure with preserved ejection fraction (HFpEF), and the diagnostic tools and therapeutic options for this population are even more limited (2, 3). There is an urgent need to further understand the pathophysiology of T2DM-associated diabetic cardiomyopathy and HFpEF in order to enable more sophisticated and targeted clinical approaches.

Known pathogenic and potentially targetable mechanisms of diabetic failing myocardium include impaired insulin metabolic signaling, intracellular calcium dysregulation, mitochondrial dysfunction, and oxidative stress (4). In nondiabetic failing cardiomyocytes, the calcium-handling microdomains at transverse-tubules (t-tubules) organized by cardiac bridging integrator 1 (cBIN1) (5) are disrupted (6, 7). These microdomains serve as local trafficking and signaling hubs for beat-to-beat calcium-transient development and diastolic calcium removal in healthy cardiomyocytes (5, 8, 9). Microdomain disruption, as occurs in heart failure, leads to weakened calcium transients and diastolic calcium overload (6, 9–11). In human and animal models of nondiabetic heart failure, myocardial cBIN1 is transcriptionally downregulated (7, 9, 12) with impaired t-tubule microdomain architecture, and restoration of cBIN1-microdomains rescues calcium cycling and cardiac function (6, 7). Altered cBIN1-microdomains in failing cardiomyocytes can also be measured from plasma samples (13) by cBIN1 score (CS) (14), an index of myocardial remodeling. However, it is not known whether microdomains are disrupted in cardiomyocytes from hearts with diabetic cardiomyopathy.

In diabetic hearts, in addition to diastolic calcium dysregulation, insulin resistance impairs glucose uptake due to decreased surface expression of glucose transporter 4 (GLUT4) (15, 16). T-tubules are known to draw GLUT4 translocation to allow for insulin-induced glucose uptake (17, 18), providing carbohydrate fuel to adjacent mitochondria. Impaired GLUT4 expression and surface translocation contributes to the pathophysiology of insulin resistance, diabetes, and impaired myocardial metabolism (16, 19, 20). The primary stimulus for GLUT4 translocation in cardiomyocytes is insulin binding to its receptors and signaling through the intracellular phosphoinositide 3-kinase pathway (16), which promotes microtubule-dependent translocation of GLUT4 to sarcolemma and the t-tubule membrane (21, 22). Given the role of cBIN1 in facilitating microtubule-dependent trafficking of calcium channels (8) and its binding affinity to phospholipids (23), it is possible that cBIN1 may modulate GLUT4 trafficking and glucose uptake in diabetic hearts. In the current study, we also explored GLUT4 translocation with respect to membrane microdomains in diabetic hearts.

## Results

### cBIN1 is downregulated in diabetic hearts, worsening exercise tolerance and blood glucose levels.

**Figure 1:**
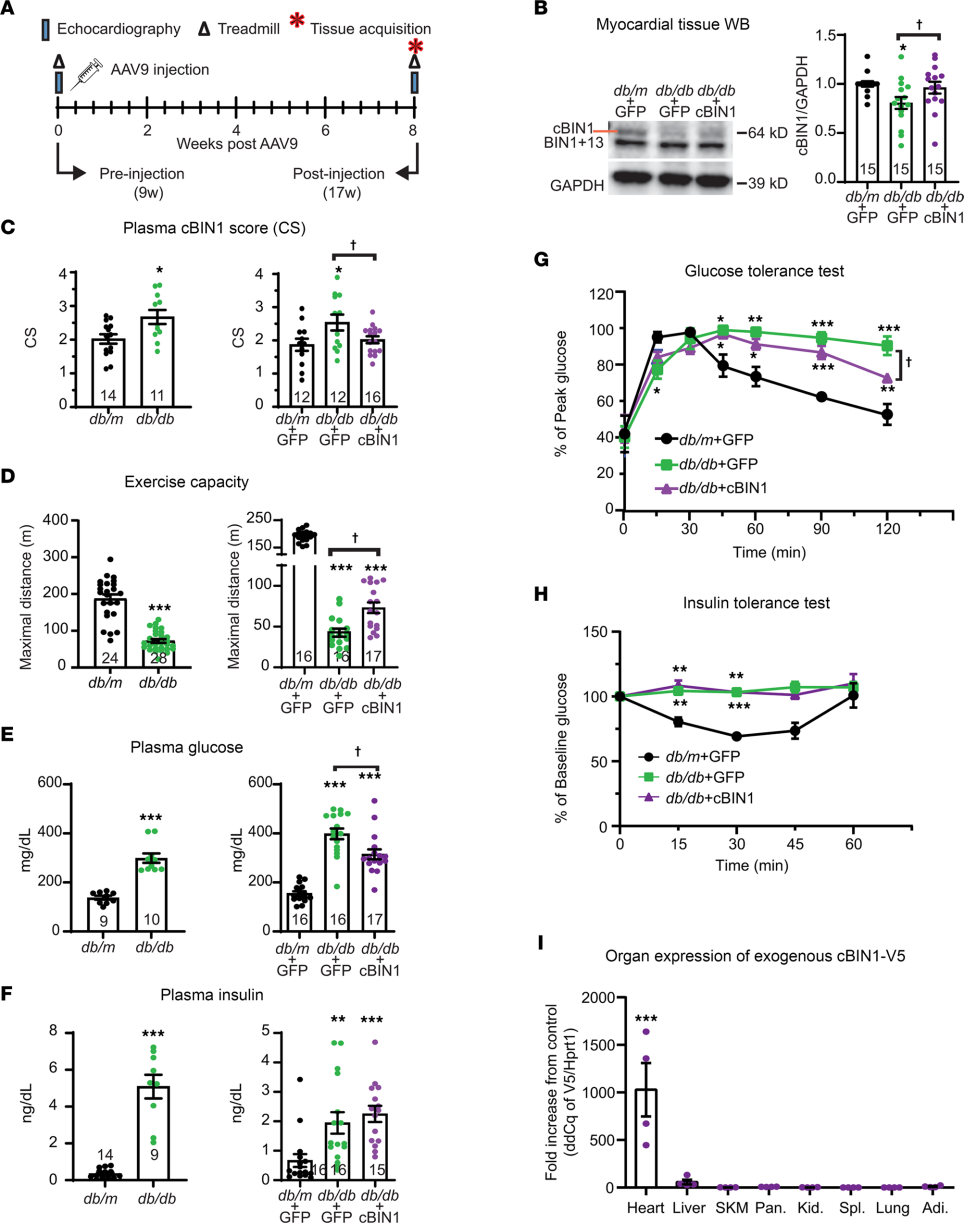
cBIN1 is downregulated in diabetic mouse hearts and can be rescued by AAV9-cBIN1 to improve exercise capacity and systemic blood glucose control. (**A**) Schematic illustration of experimental protocol used. (**B**) Western blots of cBIN1 and GAPDH with quantification (cBIN1/GAPDH) in heart lysates from posttreatment mice (*n* = 15 per group). (**C**–**F**) Bar graphs of plasma cBIN1 score (CS) values (*n* = 11–16, **C**), maximal running distance (*n* = 16–28, **D**), nonfasting plasma glucose (*n* = 9–17, **E**), and insulin (*n* = 9–16, **F**) in 9-week-old pretreatment (left) and 17-week-old posttreatment (right) db/db and littermate control db/m mice. (**G**) Percent of peak blood glucose during iGTT in AAV9-treated mice at 17 weeks of age (*n* = 4–6 animals per group). (**H**) Percent of baseline blood glucose during iITT in AAV9-treated mice at 17 weeks of age (*n* = 4–6 animals per group). (**I**) qPCR analysis of V5-tagged exogenous cBin1 (V5 normalized to the housekeeping gene Hprt1 then compared with noninjected control organs) in indicated organs from db/db mice injected with AAV9-cBIN1-V5. SKM, skeletal muscle; Pan, pancreas; K, kidney; Sp, spleen; Adi, adipose tissue; *n* = 4 animals per group. Data are presented as mean ± SEM. Unpaired 2-tailed Student’s *t* test or nonparametric Mann-Whitney *U* test was used for 2 group comparison. *, *** indicates *P* < 0.05, 0.001, respectively, for comparison versus db/m (**C**–**F**) or no virus controls (**I**). One-way ANOVA followed by Bonferroni’s test or nonparametric Kruskal-Wallis test followed by Dunn’s test was used for comparison between selected pairs (**B**–**F**). Two-way ANOVA followed by Tukey’s test was used for comparison on multiple timepoints among treatment groups (**G** and **H**). *, **, *** indicates *P* < 0.05, 0.01, and 0.001, respectively, for comparison versus db/m + GFP; ^†^ indicates *P* < 0.05 for comparison between db/db + GFP and db/db + cBIN1.

To explore cBIN1 expression in hearts with diabetic cardiomyopathy, we used leptin receptor–deficient Lepr^db^ (db/db) mice, which is a common obesity-associated T2DM model (Figure 1A). As indicated in Figure 1B, Western blotting analysis of myocardial tissue lysates identified that cardiac cBIN1 expression is near 20% (19.4% ± 6.1 %) reduced in 17-week old db/db mouse hearts when compared with lean heterozygous Dock7^m^Lepr^db^ (db/m) control hearts, which can be normalized by in vivo gene transfer of AAV9-cBIN1 transduced exogenous cBIN1 (1 ***×*** 10^11^ vector genome [vg] in 100 mL of PBS). Consistent with Western blotting results, the plasma CS (14), which is an index of myocardial remodeling and the inverse of myocardial cBIN1 expression (13), is already increased in 9-week-old db/db mice and normalizes with AAV9-cBIN1 therapy (Figure 1C). We found that, unlike control db/m littermates, db/db mice by the age of 9 weeks already developed phenotypic diabetic cardiomyopathy with impaired exercise tolerance and diastolic dysfunction, as evidenced by alterations in echocardiogram-measured diastolic parameters (reduced E/A, elevated E/e’, and prolonged IVRT, where E indicates peak velocity blood flow from left ventricular relaxation in early diastole, A indicates peak velocity flow in late diastole caused by atrial contraction, e’ indicates early diastolic mitral annular tissue velocity, and IVRT indicates isovolumic relaxation time) (Figure 1D, Supplemental Figure 1, and Supplemental Table 1; supplemental material available online with this article; https://doi.org/10.1172/jci.insight.166713DS1). Administration of AAV9-cBIN1 improved exercise tolerance in db/db mice, as indicated by increased running distance on a treadmill in cBIN1-treated db/db mice relative to AAV9-GFP treatment (Figure 1D). Body weight remained increased in db/db mice with no difference observed between GFP and cBIN1 treatment (Supplemental Table 2), which indicates that improved exercise tolerance in db/db + cBIN1 mice was independent from body weight and was most likely due to a functional recovery of cardiac hemodynamics.

To our surprise, not just exercise but elevated plasma glucose levels in 17-week-old db/db mice were also partially rescued with cBIN1 treatment (Figure 1E), as was systemic glycemic control (Figure 1G). However, plasma insulin levels and insulin tolerance capacity remained similarly impaired between GFP and cBIN1 treatment (Figures 1, F and H).

The improved glucose regulation with cBIN1 therapy suggests that cardiac muscle can affect system glucose levels. To confirm that cBIN1 transduction occurs primarily in the heart, we developed a TaqMan probe detecting the V5 sequence included only in the exogenous gene transduced by AAV9. As illustrated in Figure 1I, quantitative PCR (qPCR) analysis of cDNA samples obtained across 9 organs (heart, liver, skeletal muscle, pancreas, kidney, spleen, lung, and adipose) identified that exogenous cBIN1-V5 is only effectively transduced in hearts. Immunofluorescence labeling with anti-V5 antibody followed by spinning-disc confocal imaging detected transduction of exogenous cBIN1-V5 protein in near 100% of cardiomyocytes in db/db mouse hearts without transduction in skeletal muscle (Supplemental Figure 2). This result is in consistent with the literature because the CMV-promoted AAV9 vector is well established for cardiac tropism with retained cardiac selectivity at a relative low dose of 1 × 10^11^ vg as used in our study (24). These data indicate that improved exercise capacity and systemic glycemic control in db/db mice originated from cardiac transduction of exogenous cBIN1.

### AAV9-cBIN1 normalizes microdomains at cardiac t-tubules in diabetic heart failure.

**Figure 2:**
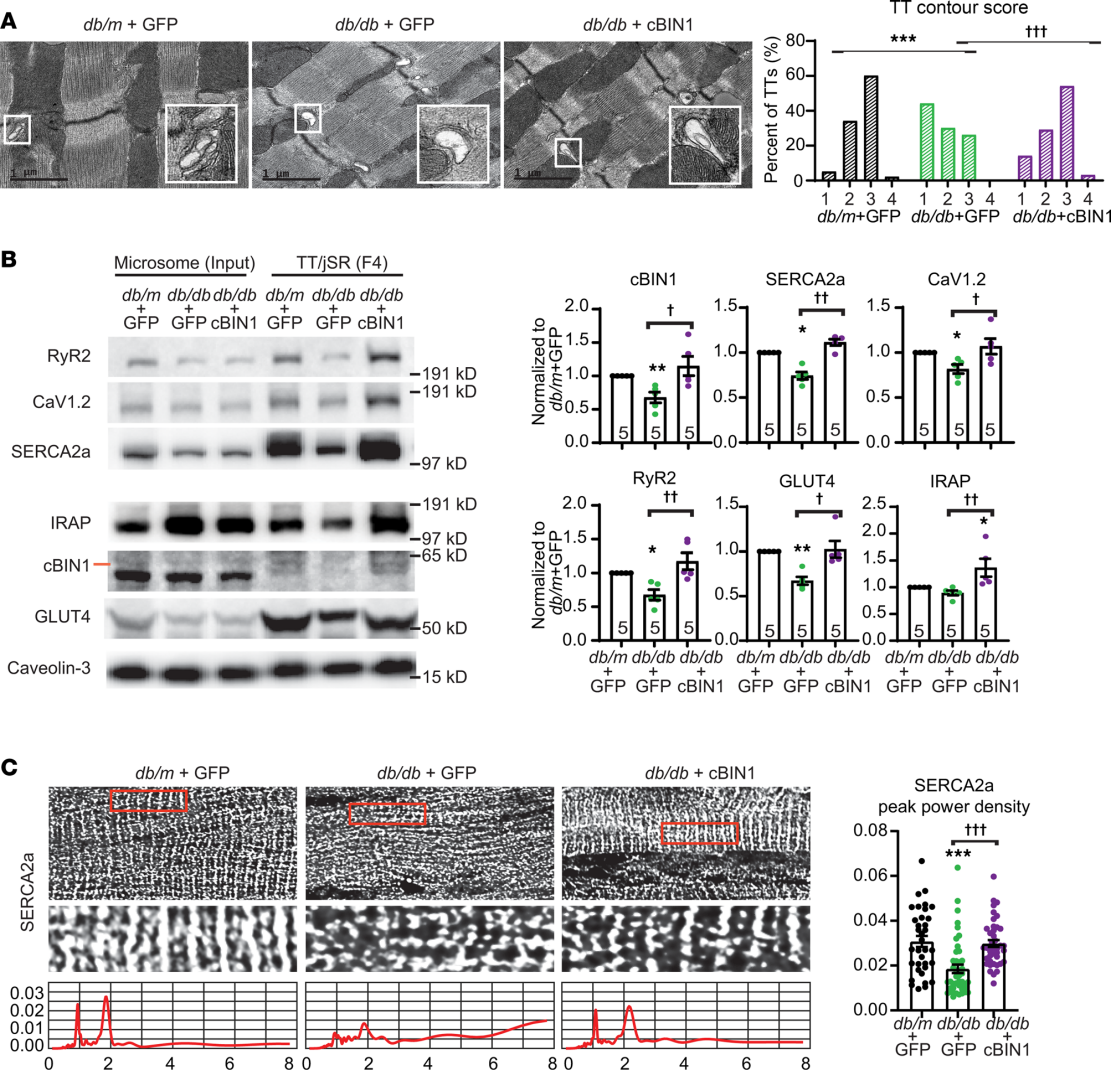
Exogenous cBIN1 normalizes membrane microdomains at t-tubules. (**A**) Representative transmission electron microscopy images of posttreatment hearts (scale bar: 1 μm) from each group (left). Quantification of the degree of contour of t-tubules (*n* = 100–101 t-tubules from 16–40 images of 2–3 myocardial sections and 3 hearts from each group). Data are presented as percentage of t-tubules. χ^2^ test was used to compare t-tubule contour between groups. *** indicates *P* < 0.001 for db/m + GFP versus db/db + GFP; ^†††^ indicates *P* < 0.001 for db/db + GFP versus db/db + cBIN1. (**B**) Western blots of cBIN1, SERCA2a, CaV1.2, RyR2, GLUT4, and IRAP in total cardiac microsome and sucrose-gradient isolated TT/jSR fraction (F4) from each group (*n* = 5 hearts per group). (**C**) Representative spinning disc confocal images of posttreatment mouse myocardium with power spectrum analysis of boxed areas, and quantification of SERCA2a peak power density at t-tubules (*n* = 35–44 cells from 3 hearts per group). Nonparametric Kruskal-Wallis test followed by Dunn’s test was used for comparison between selected pairs. *, **, *** indicates *P* < 0.05, 0.01, and 0.001, respectively, for comparison versus db/m + GFP; ^†^, ^††^, ^†††^ indicates *P* < 0.05, 0.01, 0.001, respectively, for comparison between db/db + GFP and db/db + cBIN1.

Given the capacity of cBIN1 to form t-tubule membrane microfolds (5) and that cBIN1 decreases during diabetic cardiomyopathy progression (Figure 1), we explored t-tubule membrane ultrastructure of diabetic cardiomyocytes and in response to cBIN1 gene therapy. Using transmission electron microscopy (TEM) imaging, we found that decreased cBIN1 in db/db cardiomyocytes was associated with loss of t-tubule microfolds as quantified by a t-tubule contour score (5, 6), with restoration by cBIN1 gene therapy (Figure 2A).

We next used biochemical sucrose gradient–based fractionation of cardiac microsomes (6) to evaluate subcellular distribution of proteins associated with microsomes originated from cBIN1 microfolds. The 38%/45% sucrose gradient interface (fraction 4 [F4]) had the highest cBIN1 yield, indicating cBIN1-microfolds origin. The same cBIN1-enriched microsome fraction also contained t-tubule/junctional sarcoplasmic reticulum (TT/jSR) proteins including CaV1.2 (the pore-forming subunit of L-type calcium channel [LTCC]), ryanodine receptor 2 (RyR2), and sarcoplasmic reticulum calcium-ATPase 2a (SERCA2a; Figure 2B). These data are consistent with previous results that cBIN1-microfolds house both the systolic calcium releasing unit formed by LTCC-RyR2 dyads (9) and a subpopulation of SERCA2a (6, 7, 9). In cardiac microsomes prepared from db/db hearts with reduced cBIN1, there was a significant reduction of the amount of the F4-located jSR subpopulation of SERCA2a (Figure 2B). Total SERCA2a expression was also decreased, though it can be restored with cBIN1 treatment (Supplemental Figure 3A).

Immunofluorescence imaging with power spectrum analysis further confirmed that intracellular organization of SERCA2a became disorganized in db/db mouse cardiomyocytes and was normalized by AAV9-cBIN1 gene therapy (Figure 2C). Furthermore, in db/db hearts with still-preserved systolic function, there were no significant changes in the overall myocardial expression of LTCCs and RyRs (Supplemental Figure 3A), while their concentration to the TT/jSR fraction was readily increased in response to exogenous cBIN1 treatment (Figure 2B). These data indicate that targeting cBIN1-microdomains in diabetic cardiomyocytes, similar to sympathetic-overdriven cardiomyocytes with diastolic dysfunction (6), can organize calcium handling machinery, effective both total concentration, and, to a greater extend, localization of critical proteins to t-tubule membranes.

### GLUT4 is enriched in cBIN1 microdomains.

**Figure 3:**
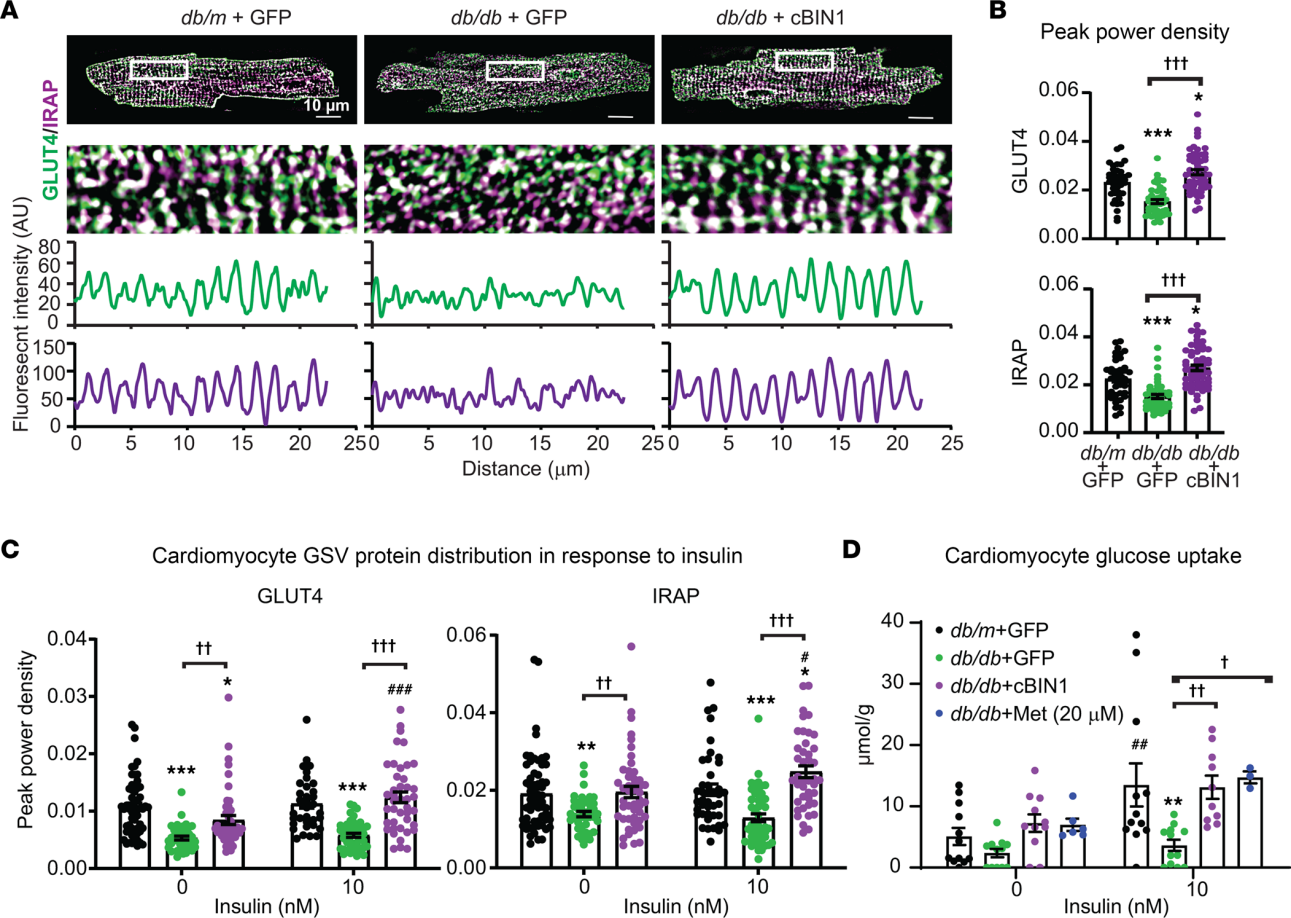
Exogenous cBIN1 normalizes GLUT4 expression at t-tubules. (**A** and **B**) Representative GLUT4 and IRAP confocal images in posttreatment isolated cardiomyocytes with corresponding fluorescence intensity profiles (**A**), and quantification of GLUT4 and IRAP peak power density (**B**) at t-tubules (*n* = 42–58 cells from 3 hearts). Scale bar: 10 μm. Data are presented as mean ± SEM. Kruskal-Wallis test followed by Dunn’s test was used for comparison between selected pairs. *, *** indicates *P* < 0.05, 0.001, respectively, for comparison versus db/m + GFP; ^†††^ indicates *P* < 0.001 for comparison between db/db + GFP and db/db + cBIN1. (**C** and **D**) Intracellular distribution of GLUT4 and IRAP (peak power density analysis, *n* = 34–61 cells from 3 hearts) (**C**) and glucose uptake (**D**) following insulin (0 and 10 nM) stimulation in cardiomyocytes isolated from each group (*n* = 10–12 repeats from 4 animals per group). Metformin (Met; 20 μM) was included as a positive control treatment. Two-way ANOVA followed by Tukey’s (3 or more treatment groups) or Bonferroni’s test (2 insulin doses) was used for multiple comparison among groups. **, *** indicates *P* < 0.01, 0.001, respectively, for comparison versus db/m + GFP within the same insulin dose groups, and ^†^, ^††^, ^†††^ indicates *P* < 0.05, 0.01, 0.001, respectively, for comparison versus db/db + GFP within the same insulin dose groups. ^#^, ^##^, ^###^ indicates *P* < 0.05, 0.01, 0.001, respectively, for comparison of 10 nM insulin versus 0 insulin baseline within each therapeutic group.

In addition to calcium-handling proteins, based on the results of Figure 1, we probed cBIN1 microsomes for GLUT4 and noted impressive enrichment in the same F4-TT/jSR fraction (Figure 2B). This enrichment raised the possibility that cBIN1 microfolds at t-tubules may also be involved in GLUT4 surface translocation upon insulin stimulation. Basally, GLUT4 is present mainly in intracellular GLUT4 storage vesicles (GSVs). Upon insulin stimulation or muscle contraction, GLUT4 is translocated to the surface membrane to enable a rapid increase in glucose uptake (21). We explored GLUT4 expression and localization in db/db cardiomyocytes in response to AAV9-cBIN1 treatment. While AAV9-cBIN1 did not alter total GLUT4 protein expression, which is slightly reduced in db/db hearts (Supplemental Figure 3B), AAV9-cBIN1 therapy significantly increased GLUT4 localization to the t-tubule membrane as indicated by the results from both biochemical fractionation (Figure 2B) and fluorescence imaging analysis of myocardial cryosections (Supplemental Figure 4A) and isolated cardiomyocytes (Figure 3, A and B).

Insulin-responsive aminopeptidase (IRAP) is responsible for GSV trafficking in insulin-sensitive tissues (21, 25). We therefore examined the protein expression and subcellular distribution of IRAP. Exogenous cBIN1 increased IRAP expression in whole-heart lysates and the TT/jSR region, as indicated by membrane fractionation and fluorescence confocal imaging results (Figure 2B; Figure 3, A and B; and Supplemental Figure 3B). Furthermore, in isolated cardiomyocytes treated with insulin for 20 minutes, there was cBIN1-mediated rescue of impaired intracellular distribution of GLUT4 and IRAP to the TT/jSR region in diabetic db/db cardiomyocytes (Figure 3C). It is also worth noting that the GLUT4 responsiveness to exogenous cBIN1 in cardiac muscle did not extend to skeletal muscle. In skeletal muscle, GLUT4 distribution was not significantly altered in db/db mice, with little change after AAV9-cBIN1 treatment (Supplemental Figure 4B).

Impaired GLUT4 expression and translocation in insulin-sensitive tissue contributes to the hyperglycemia observed in T2DM (19). To explore whether cardiac muscle can affect glucose concentration in vitro, cardiomyocytes were isolated, and after starvation, insulin-stimulated uptake of 2-Deoxy-D-glucose (2DG) was measured. The db/db + GFP cardiomyocytes had a profound loss of glucose uptake in the presence of 10 nM insulin, but uptake was normalized by AAV9-cBIN1 pretreatment. In fact, cBIN1 rescue of glucose uptake was quantitatively comparable with the rescue induced by the glucose-lowering drug metformin (20 μM), which is known to increase cell surface expression of GLUT4 in cardiomyocytes (26) (Figure 3D).

**Figure 4:**
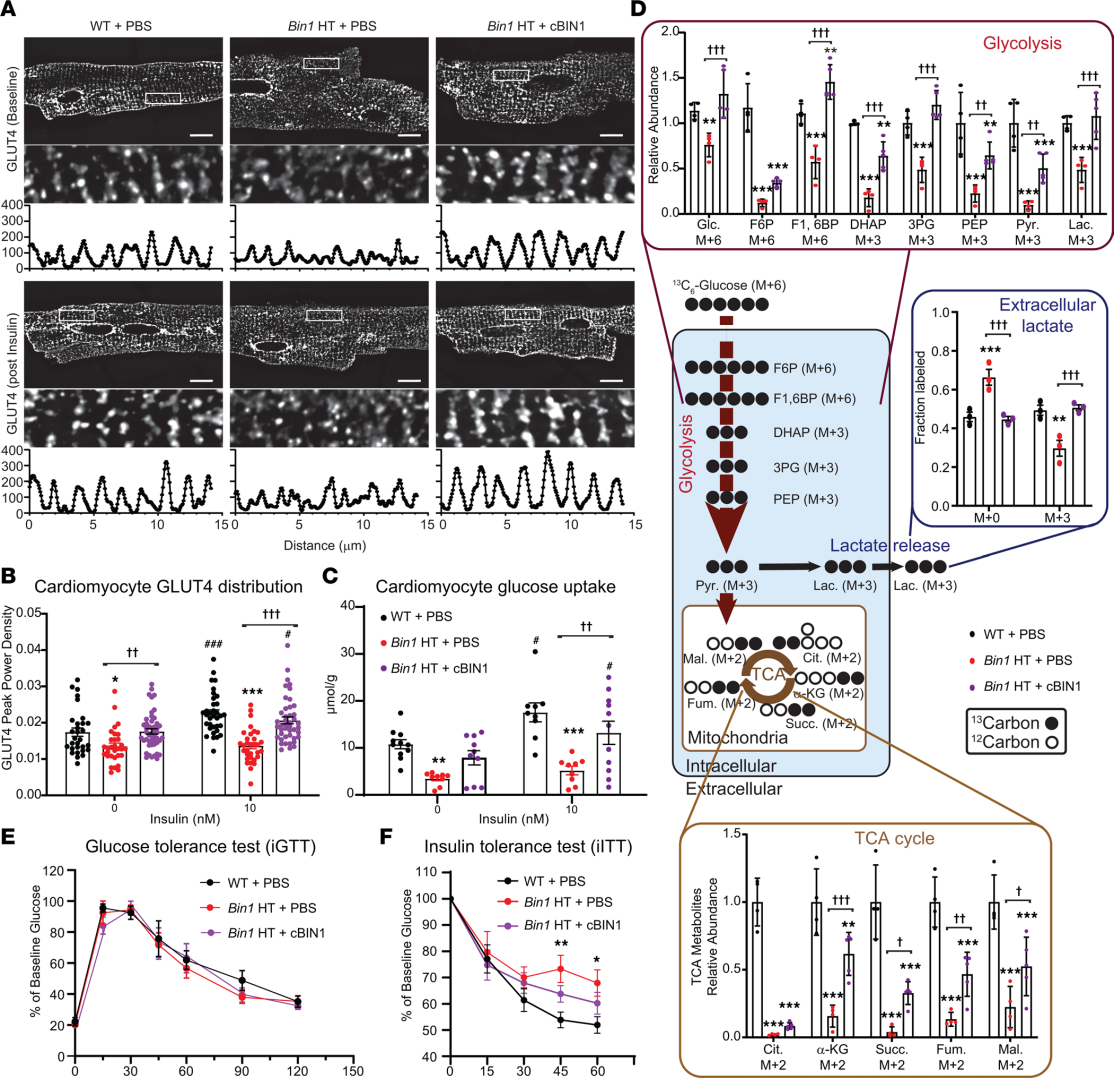
*Bin1* deletion in cardiomyocytes impairs GLUT4 translocation and glucose utilization, weakening systemic glucose response to insulin in mice. (**A**) Representative GLUT4 confocal images in isolated cardiomyocytes from WT and Bin1 HT mice with or without prior AAV9-cBIN1 rescue. Cardiomyocytes were treated with 0 or 10 nM insulin for 20 minutes before collection. The images of the boxed areas with the corresponding fluorescence intensity profiles are included in the bottom panels. Scale bar: 10 μm. (**B**) Quantification of GLUT4 peak power density at t-tubules (*n* = 30–47 cells from 3 hearts per group). (**C**) 2DG uptake following insulin (0 and 10 nM) stimulation in cardiomyocytes isolated from each group (*n* = 10–12 repeats from 3 animals per group). (**D**) ^13^C enrichment of extracellular lactate and intracellular glycolysis and TCA cycle intermediates from cardiomyocytes isolated from each group (*n* = 3–5). Schematic for metabolic pathway of ^13^C_6_-glucose is included. Pyr., pyruvate; Lac., lactate; Glc, glucose; F6P, fructose-6-phophate; F1,6BP, fructose-1,6-biphosphate; DHAP, dihydroxyacetone phosphate; 3PG, 3-phosphoglyceric acid; PEP, phosphoenolpyruvate; Cit., citrate; α-KG, alpha-ketoglutarate; Succ., succinate; Fum., fumarate; Mal, malate. (**E**) Percent of peak blood glucose during iGTT in each group (*n* = 4). (**F**) Percent of baseline blood glucose during iITT in each group (*n* = 4). Data are presented as mean ± SEM. Two-way ANOVA followed by Tukey’s (3 treatment groups, **B**–**F**) or Bonferroni’s (2 insulin doses, **B** and **C**) test is used. *, **, *** indicates *P* < 0.05, 0.01, 0.001, respectively, for comparison versus WT + PBS (**B**–**F**); ^†^, ^††^, ^†††^ indicates *P* < 0.05, 0.01, 0.001, respectively, for comparison between Bin1 HT + PBS and Bin1 HT + cBIN1 (**B**–**F**); and ^#^, ^###^ indicates *P* < 0.05, 0.001, respectively, for comparison of 10 nM versus 0 nM insulin within each group (**B** and **C**).

The GLUT4 trafficking data (Figure 3, A–C) together with improved blood glucose tolerance (Figure 1G) with reduced hyperglycemia (Figure 1E) following cBIN1 treatment in db/db mice, and ability of cardiomyocytes to take up glucose (Figure 3D), collectively point to a possibility of direct cardiomyocyte contribution to blood glucose regulation in patients with T2DM. To test the relationship between cBIN1 and GLUT4 expression without the complication of T2DM, we analyzed glucose uptake in mice with cardiac-specific heterozygous Bin1 deletion (Bin1 HT, Bin1^fl/+^; Myh6-Cre^+^). Bin1 HT mice have depleted t-tubule microfolds in cardiomyocytes (5) and are sensitive to pressure overload (6). When Bin1 HT mice are more than 1 year old, they develop a significant reduction in E/A (1.04 ± 0.05 versus WT 1.58 ± 0.15, *P* < 0.01) that can be rescued by AAV9-cBIN1 (1.72 ± 0.07, *P* < 0.01 when compared with Bin1 HT). Intracellular distribution of GLUT4 and 2DG uptake were explored in Bin1 HT cardiomyocytes (with or without prior in vivo AAV9-cBIN1 rescue) following 10 nM insulin stimulation for 20 minutes. Consistent with results from db/db mice, Bin1 HT cardiomyocytes lost organized intracellular GLUT4 distribution to the TT/jSR region (Figure 4, A and B) with a functional consequence of reduced 2DG uptake (Figure 4C), which normalized with AAV9-cBIN1 therapy. With reduced glucose uptake, Bin1 HT cardiomyocytes likely have impaired glucose oxidation, providing fuel for the mitochondrial tricarboxylic acid (TCA) cycle (cartoon in Figure 4D). We therefore tracked the glucose utilization using ^13^C_6_-glucose tracing in isolated Bin1 HT cardiomyocytes. Consistent with impaired glucose uptake, there was an overall loss of ^13^C-enrichment in intracellular glycolytic and TCA cycle intermediates from labeled glucose in Bin1 HT cardiomyocytes (Figure 4D). Furthermore, we also noted a resultant decreased release of heavy-labeled lactate (M+3) from Bin1 HT cardiomyocytes into the extracellular bathing medium (Figure 4D). With prior in vivo AAV9-cBIN1 treatment, the abnormalities in glucose tracing could be rescued. Moreover, in these Bin1 HT lean mice with still-preserved glucose tolerance (Figure 4E), in vivo insulin administration resulted in less reduction in blood glucose levels, which could be improved with prior in vivo rescue with AAV9-cBIN1 (Figure 4F). These data, together with results from db/db mice (Figure 1, Figure 2, and Figure 3), indicate that TT/jSR localized cBIN1-microdomains may serve as a trafficking destination of GSVs, contributing to systemic glycemic control.

### Altered cardiac calcium handling and glucose uptake proteomics in diabetic heart failure are restored by AAV9-cBIN1.

**Figure 5:**
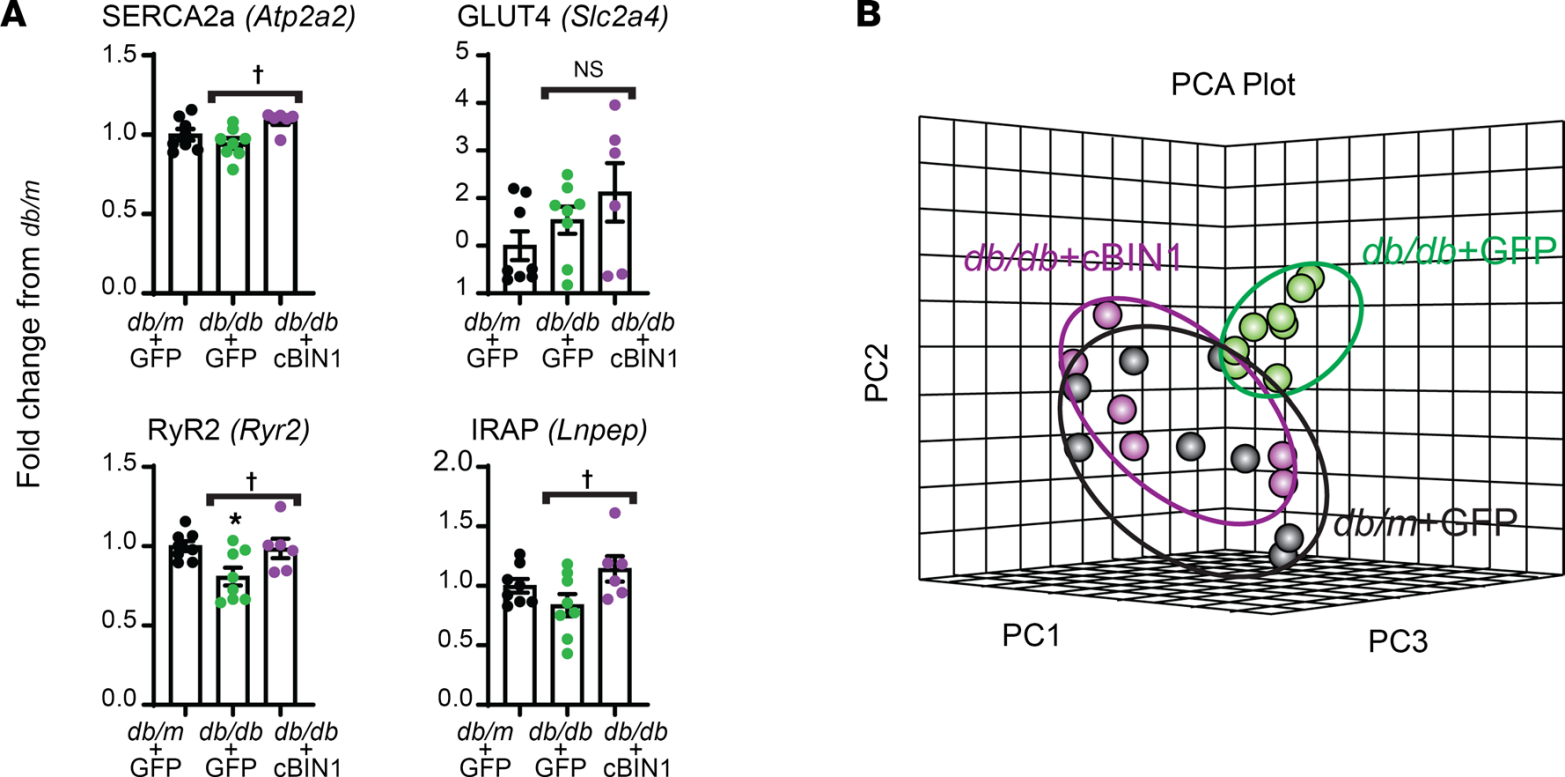
AAV9-cBIN1 normalizes cardiac proteomics in diabetic mice. (**A**) Bar graphs of LFQ LC-MS/MS data (fold changes over control db/m mice) of SERCA2a, RyR2, GLUT4, and IRAP from the db/m mice treated with AAV9-GFP (*n* = 4 hearts) and db/db mice treated with AAV9-GFP (*n* = 4 hearts) or cBIN1 (*n* = 3 hearts). (**B**) PCA plot of all 3 groups (*n* = 2 repeats/heart × 3–4 hearts/group) generated based on LFQ LC-MS/MS proteomics. All data are presented as mean ± SEM. One-way ANOVA followed by Bonferroni’s test or Kruskal-Wallis test followed by Dunn’s test was used for comparison between the selected pairs. * indicates *P* < 0.05 for comparison versus db/m + GFP; ^†^ indicates *P* < 0.05 for comparison between db/db + GFP and db/db + cBIN1.

The data of Figure 2, Figure 3, and Figure 4 indicate that cBIN1-microdomains at t-tubules served as trafficking and signaling hubs for coordinated beat-to-beat cytosolic calcium handling and glucose uptake in cardiomyocytes. To confirm proteomic recovery of diabetic myocardium in response to cBIN1 gene therapy, we performed tandem mass spectrometry–based (MS/MS-based) analysis of cardiac tissue from 8-week–postinjection mice. From these data, we identified 1,212 proteins, 93 proteins of which were affected by cBIN1 gene therapy (Supplemental Data File 1). Within these data, we then examined calcium-handling proteins (SERCA2a and RyR2) and GSV proteins (GLUT4 and IRAP). Consistent with Western blotting results, cBIN1 gene therapy resulted in a significant increase in myocardial expression of SERCA2a, RyR2, and IRAP relative to control GFP-treated db/db hearts (Figure 5A). These proteomic data (Figure 5A) support the ability of cBIN1 therapy to restore intracellular calcium handling and insulin-dependent glucose uptake in db/db cardiomyocytes (Figures 2 and 3). Additionally, as indicated in the proteomic 3-dimensional principal component analysis (PCA) plot (Figure 5B), overall protein segregation of db/db + GFP hearts from db/m + GFP hearts was close to being normalized by cBIN1 gene therapy.

### Benefits of AAV9-cBIN1 are cardiac specific.

**Figure 6:**
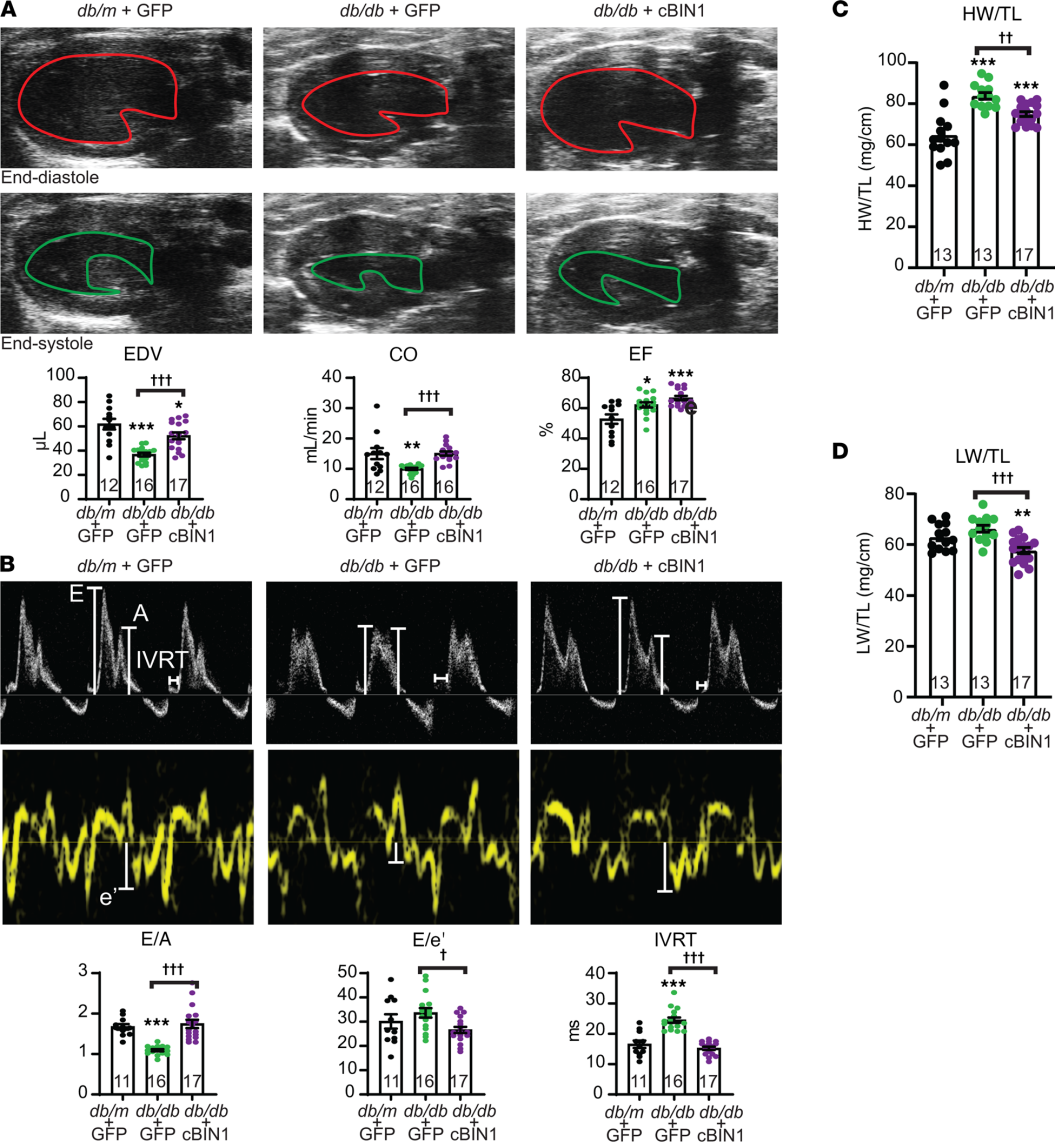
Exogenous cBIN1 rescues diastolic heart failure in diabetic mice. (**A**) Representative images of longitudinal axis view of left ventricles at end diastolic (top) and systolic (bottom) phases in posttreatment mice (17 weeks of age). Quantification of end-diastolic volume (EDV), cardiac output (CO), and ejection fraction (EF) from each group are included in the bar graphs below (*n* = 12–17). (**B**) Representative images of mitral valve inflow pulsed wave Doppler (top) and septal mitral valve annulus tissue Doppler (bottom) in posttreatment mice. Quantification of E/A, E/e’, and isovolumic relaxation time (IVRT) from each group are included in the bar graphs below (*n* = 11–17). (**C** and **D**) The ratio of heart weight (**C**) and lung weight (**D**) over tibial length after treatment (*n* = 13–17). All data are presented as mean ± SEM. One-way ANOVA followed by Bonferroni’s test or Kruskal-Wallis test followed by Dunn’s test was used for comparison between selected pairs. *, **, *** indicates *P* < 0.05, 0.01, 0.001, respectively, for comparison versus db/m + GFP; ^†^, ^††^, ^†††^ indicates *P* < 0.05, 0.01, 0.001, respectively, for comparison between db/db + GFP and db/db + cBIN1.

In Figure 1, we found that cBIN1 therapy improved exercise tolerance, as well as glucose and insulin tolerance, in diabetic mice. We next explored the details of the functional rescue. Posttreatment echocardiograms were obtained from 17-week-old mice, with AAV9 administered 8 weeks earlier. Echocardiography provides measurement of cardiac geometry, wall thickness, and left ventricular contractile and diastolic function. As indicated in the representative 2-dimensional long-axis–view images of the left ventricles (Figure 6A), db/db mouse hearts had an impaired diastolic phenotype with a significant concentric hypertrophy, as evidenced with increased wall thickness and reduced ventricular volume (Supplemental Table 2) yet with preserved ejection fraction. With AAV9-cBIN1 treatment, reduced left ventricular end diastolic volume (EDV), stroke volume (SV), and cardiac output (CO) in db/db mice were all normalized (Figure 6A and Supplemental Table 2). For diastolic functional analysis, Figure 6B contains representative pulse-wave Doppler images of E and A waves (top panel images) and tissue Doppler images of e’ velocity (bottom panel images). Diastolic parameters E/A, E/e’, and IVRT are included in the bar graphs at the bottom. When compared with db/m mice, db/db mice display a significant decrease in E/A ratio, prolongation of IVRT, and impairment of the myocardial performance index (MPI) (Figure 6B and Supplemental Table 2). AAV9-cBIN1 treatment also rescued E/A and MPI, shortend IVRT, and significantly reduced E/e’ in db/db mice. Postmortem analysis identified that the elevated heart weight [HW] and lung weight (LW; expressed as ratio to tibial length [TL]; HW/TL, LW/TL) of db/db mice were both reduced with cBIN1 treatment (Figure 6, C and D), indicating that cBIN1 gene therapy improved diabetic cardiomyopathy–associated cardiac hypertrophy and lung edema. Moreover, in 8-month-old db/db mice with worsening diabetic cardiomyopathy, we further confirmed that AAV9-cBIN1 gene therapy improved cardiac function and exercise intolerance (Supplemental Table 3).

**Figure 7:**
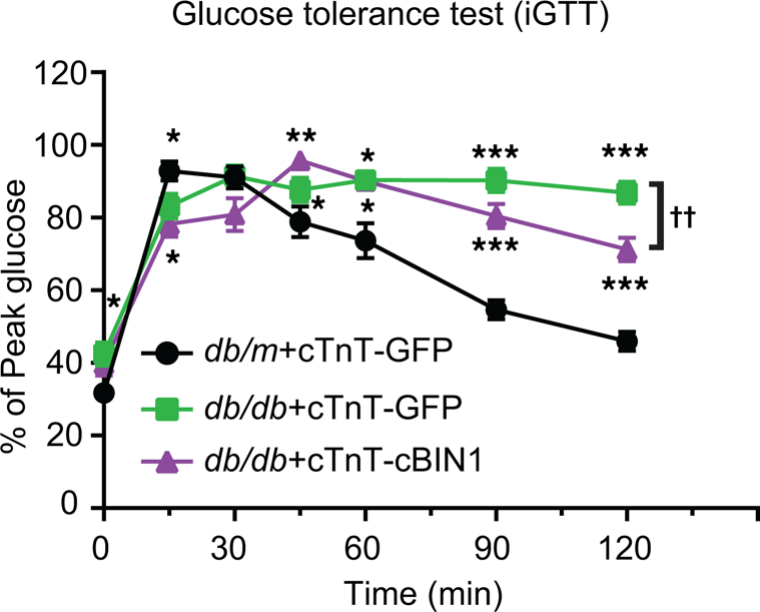
AAV9-cTnT-cBIN1 improves systemic glucose tolerance in diabetic mice. Percent of peak blood glucose during iGTT in AAV9-cTnT-GFP/cBIN1–treated mice following an i.p. injection of glucose (1 g/kg) after 12-hour fasting (*n* = 10 mice per group). Data are presented as mean ± SEM. Two-way ANOVA followed by Tukey’s for multiple comparisons among groups is used. *, **, *** indicates *P* < 0.05, 0.01, 0.001 for comparison versus db/m + GFP; ^††^ indicates *P* < 0.01 for comparison between db/db + GFP and db/db + cBIN1.

To test for cardiac specificity of the glucose response, we then applied gene therapy with a vector that drives cBIN1 expression by the cardiac-specific promotor chicken cardiac troponin T (cTnT) (27), and we repeated the functional analysis in db/db mice (*n* = 12 per group). As indicated in Figure 7, AAV9-cTnT-cBIN1 treatment in db/db mice resulted in an increased ability to clear a 1 g/kg glucose load, recapitulating the glycemic control effect induced by low-dose AAV9-CMV-cBIN1 (Figure 1G). Furthermore, following the same in vivo gene therapy protocol illustrated in Figure 1A, we identified that AAV9-cTnT-cBIN1 (1 × 10^11^ vg) also provided functional rescue of exercise capacitance and cardiac functional parameters in db/db mice (Supplemental Table 4). Together, these data suggest that cardiomyocyte membrane microdomains, by facilitating GLUT4 translocation to t-tubules, are critical to insulin-stimulated glucose uptake. Beyond cardiac hemodynamic protection, systemic glycemic control also benefited from cBIN1 gene therapy in the heart.

### I.v. AAV9-cBIN1 transduces exogenous cBIN1 in large animal hearts.

**Figure 8:**
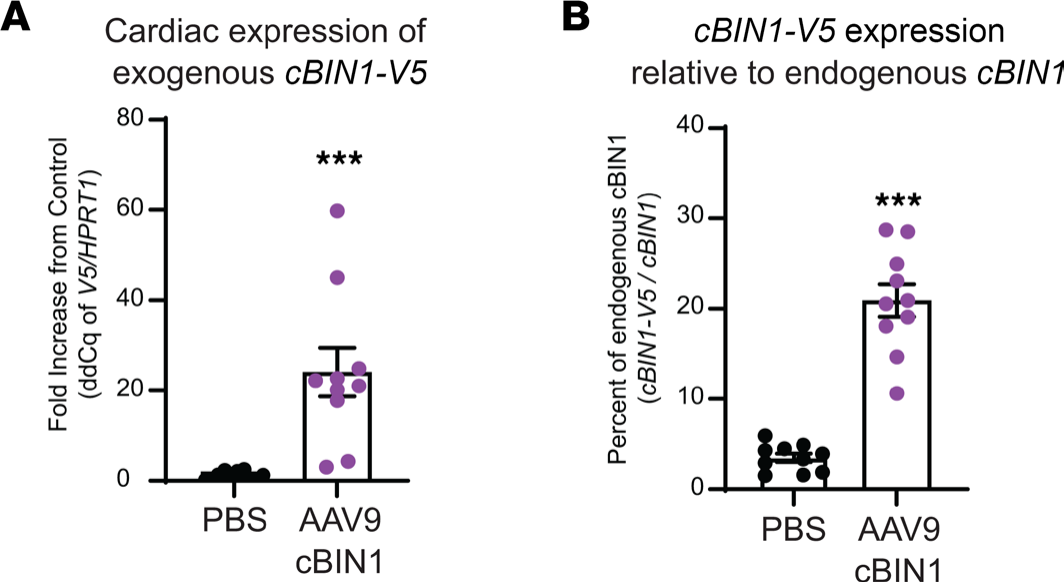
I.v. AAV9-cBIN1 (6 × 10^11^ vg/kg) transduces exogenous cBIN1 in minipig hearts. (**A**) Cardiac expression of exogenous cBin1-V5 (ΔΔCt of V5/HPRT1 when compared with PBS controls) in minipig hearts 6 months after AAV9-cBIN1 injection. (**B**) Exogenous cBin1-V5 as percentage of endogenous porcine cBIN1 (derived from ΔCt of V5/cBIN1) in minipig hearts 6 months after AAV9-cBIN1 injection (*n* = 10 tissue samples across left ventricle obtained from 2 minipigs per group). Data are presented as mean ± SEM. Unpaired 2-tailed Student’s *t* test or Mann-Whitney *U* test was used. *** indicates *P* < 0.001 for comparison versus PBS controls.

The preceding data indicate a mechanistic role for cBIN1-organized cardiac microdomains in the hemodynamic and glycemic recovery of diabetic mice. To explore the translational possibility of cBIN1 gene therapy, we tested cardiac transduction efficiency of AAV9-cBIN1 in a preclinical large-animal minipig model. We introduced a single i.v. injection of low-dose — i.e., 6 × 10^11^ vg/kg — AAV9-cBIN1. This dose is similar to the AAV dose used in a hemophilia B gene therapy trial in humans (28). Six months after injection, animals were terminated and myocardial tissues across left ventricles (apex, anterior wall, posterior wall, base, and septum) were obtained for mRNA extraction and cDNA preparation. Using qPCR with the same V5 Taqman probe used for cBin1-V5 detection in mouse hearts (Figure 1I), we were able to confirm that exogeneous cBin1-V5 expression can be successfully transduced by AAV9-cBIN1 in minipig hearts. Using the ΔΔCt results of V5/HPRT1 compared with control samples, we found that AAV9 transduced exogenous cBIN1-V5 expressed 24.1-fold ± 5.4-fold above background, relative to control PBS-treated animal hearts (Figure 8A), indicating that a single i.v. therapy can last at least 6 months in cardiomyocytes.

We next quantified exogenous transduced cBin1 expression versus endogenous cBIN1 expression. A TaqMan probe was designed to detect only the exonal junction sequence between porcine BIN1 exons 13 and 17 (cospliced in cBIN1) without recognition of the exogenous mouse cBin1 sequence transduced by AAV9. Using the ΔCt results of V5/cBIN1 (porcine), we determined that, at 6 months after injection, exogenous cBin1-V5 was expressed in minipig hearts at 20.9% ± 1.8 % of the endogenous cBIN1 transcript levels (Figure 8B). Together with the observed 20% reduction in endogenous cBIN1 expression in db/db hearts (Figure 1B), these results indicate that the current low-dose systemic delivery of AAV9-cBIN1 can effectively transduce exogenous cBIN1 at a level sufficient to replace a 20% reduction of endogenous cBIN1 expression in failing hearts. Note that this transduction-efficient dose of AAV9-cBIN1 (6 × 10^11^ vg/kg, i.v.) is close to the lowest therapeutic effective doses tested in phase I/II trials (29, 30) and is only 1%–3% of the doses used for phase III trials (31, 32) for AAV-mediated gene therapies for other diseases. We therefore could achieve cardiac transduction in a large-animal using doses of virus that are sufficiently low to limit off-target and potentially toxic side effects.

## Discussion

In this study, we identified that, in diabetic mice, cBIN1 gene therapy can restore cardiomyocyte t-tubule microdomains, normalizing cardiomyocyte subcellular architecture. This approach can help rescue diastolic dysfunction and clinical features of HFpEF and can also rescue a deficit in glucose uptake in diabetic cardiomyocytes. Furthermore, AAV9-cBIN1 gene therapy can improve hyperglycemia in the T2DM mice. In a large-animal minipig model, i.v. introduction of even lower-dose AAV9-cBIN1 provides sufficient cardiomyocyte transduction to compensate for loss of cBIN1 in diabetic heart failure.

cBIN1-microdomains are responsible for proper intracellular trafficking and organization of calcium handling machinery (5, 9, 10), and disruption in cBIN1-microdomain contributes to heart failure pathophysiology (9, 10). In murine heart failure induced by chronic pressure overload, cBIN1 gene therapy is effective in normalizing microdomains to restore intracellular localization of systolic calcium handling dyads to the TT/jSR regions, resulting in functional rescue of systolic failure (6). Moreover, in mice with sympathetic overdrive resulting in left ventricular hypertrophy and diastolic dysfunction, AAV9-cBIN1 reorganizes the diastolic calcium regulator SERCA2a to normalize cardiac lusitropy (7). Taken together, cBIN1 microdomains are a critical trafficking hub for both systolic and diastolic calcium-handling proteins, suggesting that preservation of cBIN1 microdomains could benefit diabetic failing hearts as well. In the current db/db mice with diabetic heart failure without left ventricular dilation, myocardial cBIN1 was only 20% reduced (Figure 1B), which was enough to impair membrane microdomains without affecting t-tubule lumen size. With disease progression of diabetic cardiomyopathy, cBIN1 may further decline and manifest with t-tubule lumen enlargement as occurring in nondiabetic dilated cardiomyopathy (5, 9, 10). Nevertheless, just 20% cBIN1 reduction and impairment of microdomains was already enough to cause diastolic dysfunction (Figure 6). cBIN1 gene therapy improved lusitropy and normalized EDV in db/db hearts, rescuing CO and improving exercise intolerance (Figures 1 and 6).

Our data also indicate, to our surprise, that cBIN1-microdomains at t-tubules could modulate cardiomyocyte glucose uptake by supporting GLUT4 trafficking and surface translocation (Figure 2, Figure 3, Figure 4). There is increasing evidence that GLUT4 is an important regulator of cardiac metabolism in diabetes (33, 34). Furthermore, by improving insulin-stimulated surface translocation of GLUT4 in cardiomyocytes, AAV9-cBIN1 helped rescue hyperglycemia in db/db mice (Figures 1 and 7).

The heart is not normally considered a major contributor to modulating blood glucose levels, yet even cardiac-specific expression of cBIN1 reduced hyperglycemia (Figure 7). Furthermore, data from isolated adult mouse cardiomyocytes indicate that cBIN1 promoted insulin-stimulated glucose uptake by db/db cardiomyocytes, an effect quantitatively similar to that induced by metformin treatment (Figure 3D). The role of cBIN1 microdomains in regulating insulin-stimulated cardiomyocyte glucose utilization was further supported by impaired intracellular GLUT4 trafficking, 2DG uptake, and ^13^C_6_-glucose uptake and oxidation in Bin1 HT cardiomyocytes together with rescue by AAV9-cBIN1 (Figure 4, A–D). In db/db mice with T2DM with already-elevated plasma insulin levels, cBIN1-increased cardiomyocyte insulin sensitivity suggests that heart muscle served as a tissue clearance pathway for systemic glucose control. The altered blood glucose response following insulin administration in aged Bin1 HT mice with a cardiac-specific deletion of Bin1 (Figure 4) further supports a possible role of insulin-stimulated cardiomyocyte glucose uptake in regulation of systemic glycemic control. Future studies are needed to elucidate the potential use of cBIN1 gene therapy to lower blood glucose in T2DM. Given that skeletal t-tubules express endogenous BIN1 (23, 35) and that insulin-stimulated GLUT4 translocation increases glucose utilization in skeletal muscle (36), it will be compelling to explore whether skeletal muscle transduction of cBIN1 or the equivalent skeletal isoform of BIN1 provides additional benefit in hyperglycemic control in T2DM.

The importance of cBIN1 microdomains in diabetic diastolic function and overall glycemic control has obvious therapeutic implications. Because cBIN1 is a large intracellular protein, introduction of exogenous cBIN1 in cardiomyocytes requires gene therapy. In our minipig study, low-dose cBIN1 both potentially normalized reduced cBIN1 levels in failing hearts (Figure 1 and Figure 8B) and maintained expression for at least 6 months (Figure 8). Much of the toxicity of AAV-based gene therapy is dose dependent and associated with hepatic dysfunction (37, 38). Low-dose therapy, such as that less than 1 × 10^12^ vg/kg, offered significant potential to translate cBIN1 therapy to humans (Figure 8) (28, 39). In general, introducing a trafficking-related protein can achieve a desired phenotype yet at a low dose. For instance, a low-dose introduction of GJA1-20k can reduce ventricular arrhythmia in mouse models of arrhythmogenic cardiomyopathy (40). Trafficking-related proteins may be attractive targets for multiple gene therapy solutions to cardiac disease. Future therapeutic efficacy studies in preclinical large-animal models could be a gateway to evaluate the therapeutic potential of exogenous cBIN1 for patients with T2DM and diabetic cardiomyopathy.

In conclusion, our data indicate that cBIN1-microdomains at t-tubules serve as a multifunctional hub modulating cardiomyocyte function and glycemic control. Future mechanistic studies will be helpful to further elucidate how cBIN1 regulates GSV trafficking and glucose uptake, as large-animal translational studies begin to bridge this work to human application.

## Methods

### Animals and ethical approvals.

Equal amount of male and female C57BLKS/J genetic background BKS.Cg-Dock7^m^ +/+ Lepr^db^/J mice (The Jackson Laboratory) were used for this study. Mice homozygous for the spontaneous point mutation in the gene encoding the leptin receptor Lepr^db^ (db/db) manifest metabolic abnormalities including obesity, dyslipidemia, and T2DM (41, 42). The lean heterozygous Dock7^m^ +/+ Lepr^db^ littermates (db/m) served as the control group, which can be identified from homozygotes before the phenotype becomes severe. In addition, adult male and female mice with cardiac-specific Bin1 heterozygote (HT) deletion (Bin1^fl/+^; Myh6-Cre^+^) with their WT (Bin1^+/+^; Myh6-Cre^+^) littermates were used (5). Mice were kept at less than 5 mice/cage in temperature-controlled cages (20°C–22°C) with a 12:12 light-dark cycle and free access to water and food. For the minipig study, 5- to 7-month-old male and female Yucatan minipigs were purchased from S & S Farms and housed in their housing pen in the comparative medicine center at the University of Utah.

### Generation and administration of AAV9.

Nine-week-old male and female db/db mice and littermate control db/m mice were randomized to receive 100 μL of 1 × 10^11^ vg of AAV9 transducing V5-tagged GFP or cBIN1 (*n* = 16–17 per group) or an equal volume (100 mL) of PBS (*n* = 5 per group) via retro-orbital injection after anesthesia (1% isoflurane in oxygen) (43). Eight weeks after injection, 17-week-old mice were terminated for further experiments. The rationale to use AAV9-CMV vectors for cardiac tropism expression, GFP as a negative control virus, viral administration route, and the detailed viral preparation method (custom produced at Welgen Inc.) were described in detail in our previous study (6). To increase the cardiomyocyte transduction efficacy, we increased the viral dose from the previously used 3 × 10^10^ vg/animal, which induced transduction in around 60% of cardiomyocytes (6), to the currently used dose of 1 × 10^11^ vg/animal, which was previously reported to be effective in transducing 81.2% of cardiomyocytes with limited transduction in liver (5.3%) and skeletal muscle (3.2%). Using anti-V5 labeling for exogenous proteins (GFP-V5 or cBIN1-V5), we identified that a single retro-orbital administration of 1 × 10^11^ vg/animal AAV9-CMV-GFP or cBIN1 was able to transduce 100% of cardiomyocytes at 8 weeks after viral injection (Supplemental Figure 2). A similar protocol was repeated in older db/m or db/db mice at 6 months of age that were injected with AAV9-CMV-GFP/cBIN1 (1 ***×*** 10^11^ vg, retro-orbital injection) and evaluated for echocardiography parameters and treadmill performance 8 weeks later at 8 months of age. The mice were then terminated. In addition to CMV promoter used for most of the current study, we also created AAV9-cBIN1/GFP-V5 using an AAV9 vector driven under the previously established cardiac-specific promoter cTnT (27) (custom produced at Welgen Inc.). The treatment protocol, dosage, and administration route of AAV9-cTnT-cBIN1/GFP-V5 remain the same as those used for the AAV-CMV viruses. In brief, 9-week-old male and female db/m and db/db mice received a dose of 1 × 10^11^ vg/animal AAV9-cTnT-GFP or cBIN1-V5 via retro-orbital injection (*n* = 12 mice per group) and were terminated 8 weeks later at 17 weeks old. The same AAV9 injection protocol was used for studies involving the cardiac-specific Bin1 HT mice line. In brief, 13-month-old male and female Bin1 HT mice and their WT littermate controls were administered with AAV9-CMV-cBIN1 (1 × 10^11^ vg) and were terminated 8 weeks later for i.p. glucose tolerance test (iGTT) and i.p. insulin tolerance test (iITT) before being subjected to cardiomyocyte isolation for imaging and functional analysis. For minipig studies, a single dose of i.v. AAV9-CMV-cBIN1-V5 (6 × 10^11^ vg/kg) was administered to minipigs via the ear vein injection. Six months after injection, animals were terminated and heart tissue was obtained for mRNA extraction and cDNA preparation.

### Experimental protocol.

All mice receiving AAV9 were terminated 8 weeks after viral injection at 17 weeks of age (Figure 1A). Echocardiography, plasma CS values, and exercise tolerance were obtained before and 8 weeks after AAV9 injection. I.p. glucose/ITTs were performed in fasting mice prior to termination. Gross physiological parameters including body weight, HW and LW, and TL were obtained at termination. Hearts were processed for TEM imaging, biochemical analysis including t-tubule fractionation, or MS analysis.

### Echocardiography.

In vivo echocardiography was obtained using a Vevo-3100 ultrasound system (Visual Sonics) as previously described (6). M-mode images in left ventricle short axis view at the proximal level of papillary muscles were used for measurement of left ventricle internal diameter, anterior and posterior wall thickness, left ventricle mass, and relative wall thickness. Parasternal left ventricle long axis view of 2-dimensional image was used for measurement of volume related parameters by tracing the left ventricle wall movement, followed by automatic calculation by the Vevo Software (Visual Sonics). Analysis of 3–5 cardiac cycles was used to generate the average result for each parameter. Doppler echocardiography in an apical 4-chamber view was obtained for diastolic functional analysis. Transmitral flow velocity (E, A) was obtained by pulse-wave Doppler, placing the sample volume at the mitral leaflet tips. Wall velocity (e’) was obtained by tissue Doppler, placing the sample volume at the septal mitral annulus.

### Exercise tolerance test.

Exercise capacity was estimated in db/m and db/db mice before and after AAV9 treatment at 9 weeks and 17 weeks of age. This test was completed to unmask cardiac abnormalities that might not be sufficient to limit an oxygen supply-demand relationships at rest but that could prevent oxygen delivery from meeting heightened myocardial oxygen demand during dynamic exercise — i.e., treadmill running (44). The current exercise tolerance test followed an established protocol with minor adjustments (45). To familiarize the mice with treadmill running, the mice being tested ran on the treadmill (Columbus Instruments) at a 20 degree incline × 5 m/min for 10 minutes per day for 3 days (46). At least 24 hours following the final acclimation period, mice ran at a 20 degree incline × 5 m/min for 6 minutes, 7 m/min for 3 minutes, 9 m/min for 3 minutes, 11 m/min for 3 minutes, 13 m/min for 3 minutes, 15 m/min for 3 minutes, and 17 m/min until the animal could no longer maintain the pace with the treadmill belt for > 10 consecutive seconds. The first 3 minutes were used for animal acclimatization to the treadmill at the day of experiment; the distance during this period is not included in data collection. Maximal running distance was recorded during the test.

### TEM.

After sacrifice, mouse coronary artery was retrogradely perfused through the aorta with 20 mL of ice-cold fixative (formaldehyde/glutaraldehyde 2.5% in 0.1M Sodium Cacodylate Buffer). All fixatives and chemicals used for TEM are from Electron Microscopy Sciences. After perfusion, left ventricles were sliced into 1 mm^3^ tissue sections, stored at 4°C in fixative, and sent to the Electron Microscopy Laboratory at University of Utah for further processing. After postfixation with 2% osmium tetroxide and prestaining with uranyl acetate, the ventricular tissue slices were dehydrated in graded ethanol and pure acetone, imbedded with epoxy resin, and sectioned at 70 nm using an ultramicrotome (Leica). Sections were poststained with acetate and lead citrate before imaging with JEM-1400Plus or JEM1200-EX (JEOL) transmission electron microscope with a CCD Gatan camera. We graded the degree of t-tubule contour using a modified scoring system reported previously (5).

### Plasma collection and component analysis CS and myocardial tissue cBIN1 analysis.

All plasma samples from mice anesthetized with isoflurane were collected from the submandibular vein with anticoagulant EDTA. Whole blood samples were centrifuged at 2,250*g* for 20 minutes at 4°C. Plasma was separated and aliquoted before being flash frozen with dry ice and ethanol. Plasma aliquots were stored in a –80°C freezer before assays.

The plasma CS (14), the natural log of the ratio of median plasma [cBIN1] in a normal human population to the measured [cBIN1], was determined following the manufacturer’s instructions of the CS Quantification ELISA kit (Sarcotein Diagnostics).

Plasma levels of glucose and insulin were determined using the Ultra Sensitive Mouse Insulin ELISA Kit (Crystal Chem) and the Mouse Glucose Assay Kit (Crystal Chem), respectively.

### Adult primary cardiomyocyte isolation, culture, and glucose uptake test.

Adult mouse cardiomyocytes were isolated from both male and female mice with collagenase II (2 mg/mL, Worthington Biochemical Corporation) using a previously described method (8, 47). Freshly isolated cardiomyocytes were seeded in laminin-precoated culture dishes and cultured in perfusion buffer (120.4 mM NaCl, 14.7 mM KCl, 0.6 mM KH_2_PO_4_, 0.6 mM Na_2_HPO_4_, 1.2 mM MgSO_4_ ***×*** 7H_2_O, 10 mM Hepes, 4.6 mM NaHCO_3_, 30 mM taurine, and 5.5 mM glucose) for 1 hour in 37°C and 5% CO_2_ incubator prior to further experiments.

For glucose uptake assay, freshly isolated cardiomyocytes were seeded and cultured in laminin-precoated 96-well plates for 1 hour prior to the test. Cells were then washed with glucose-free perfusion buffer (120.4 mM NaCl, 14.7 mM KCl, 0.6 mM KH_2_PO_4_, 0.6 mM Na_2_HPO_4_, 1.2 mM MgSO_4_ ***×*** 7H_2_O, 10 mM HEPES) twice and starved in the starvation buffer (glucose-free perfusion buffer containing 1 mM pyruvate, 0.1% BSA) for 40 minutes. Metformin (0 or 20 μM) was loaded together with starvation buffer in the indicated groups. Cells were then stimulated in the absence or presence of insulin (0, 10 nM) (Sigma-Aldrich) for 20 minutes. Following manufacture’s instruction, 2DG uptake in cardiomyocytes was then determined by Glucose Uptake Assay kit (Abcam) and quantified with a FlexStation 3 plate reader (Molecular Devices).

### [U-^13^C_6_]-glucose tracing and liquid chromatography–MS (LC-MS) analysis of polar metabolites.

The metabolites were extracted from isolated primary cardiomyocytes as previously described (48–50). Briefly, the cardiomyocytes were plated on laminin-coated 6 cm dishes in biological triplicates. Cells were initially plated on standard culture medium and washed with sterile PBS. Culture medium in which glucose was replaced by [^13^C_6_]-L-glucose (Cambridge Isotope Laboratories) was then added to the cells. Cells were allowed to grow in labeled media for 4 hours to reach steady state. Then, medium was rapidly aspirated (^13^C-labeled media were collected), and cells were washed with cold 0.9% saline on ice. In total, 3 mL of extraction solvent (80% methanol/water, precooled to −80°C) was added to each well, and the dishes were transferred to −80°C for 15 minutes. Cells were then scraped into the extraction solvent on dry ice. The supernatant of each sample was then vortexed a couple of times (30 seconds each). All metabolite extracts were centrifuged at 20,000*g* at 4°C for 10 minutes. Each sample was then transferred to a new 1.5 mL tube. Finally, the solvent in each sample was evaporated in a Speed Vacuum (Savant UVS450, Thermo Fisher Scientific) and stored at −80°C until they were run on the QExactive HF orbitrap mass spectrometer (Thermo Fisher Scientific). Extracted peak intensities were corrected for naturally occurring ^13^C isotope abundance before analysis (51, 52).

Extracted polar metabolite samples were analyzed by LC-MS. Separation was achieved by hydrophilic interaction LC (HILIC) using a Vanquish HPLC system (Thermo Fisher Scientific). The column was an Xbridge BEH amide column (2.1 mm ***×*** 150 mm, 2.5 μM particular size, 130 Å pore size, Waters Co.) run with a gradient of solvent A (20 mM ammonium hydroxide, 20 mM ammonium acetate in 95:5 acetonitrile/water, pH 9.5) and solvent B (100% acetonitrile) at a constant flow rate of 150 μL/min. The gradient function was: 0 minutes, 90% B; 2 minutes, 90% B; 3 minutes, 75% B; 7 minutes, 75% B; 8 minutes, 70% B; 9 minutes, 70% B; 10 minutes, 50% B; 12 minutes, 50% B; 13 minutes, 25% B; 14 minutes, 25% B; 16 minutes, 0% B; 20.5 minutes, 0% B; 21 minutes; 90% B; and 25 minutes, 90% B. Autosampler temperature was 4°C, column temperature was 30°C, and injection volume was 3 μL. Samples were injected by electrospray ionization into a QExactive HF orbitrap mass spectrometer (Thermo Fisher Scientific) operating in negative ion mode with a resolving power of 120,000 at *m/z* of 200 and a full scan range of 75–1,000 *m/z*. Data were analyzed using the EL-MAVEN software package, and specific peaks were assigned based on exact mass and comparison with known standards (53).

### Cardiac microsome preparation and sucrose gradient fractionation.

Microsome sucrose gradient fractionation was prepared according to an established protocol with modifications (54). Frozen hearts were minced in 2 mL ice-cold homogenization buffer (20 mM Tris [pH 7.4], 250 mM sucrose, 1 mM EDTA supplemented with HALT protease inhibitor). After homogenization with a Polytron Handheld homogenizer (low speed; 15,000 rpm), lysates were centrifuged at 12,000*g* for 20 minutes at 4°C and the supernatant (S1) was collected and kept on ice. The Pellet was then resuspended in 1 mL of the same homogenization buffer, homogenized, and centrifuged again at 12,000*g* for 20 minutes at 4°C to prepare the supernatant (S2). Microsomal supernatants were then combined (S1 + S2) and subjected to ultracentrifugation in a fixed angle rotor Ti 50.2 at 110,000*g* for 2 hours at 4°C using with Beckman Ultracentrifuge L8-70M (Beckman Coulter). After ultracentrifugation, the pellet was weighed and resuspended in the appropriate amount of homogenization buffer (~0.5 mL) to bring a final concentration of microsome to 4 mg/mL. The same amount of total microsome from each heart sample (~1.5 mg in 0.5 mL, protein concentration determined by BCA assay) was laid over the top of the prepared sucrose gradient (2 mL for 27%, 2 mL for 32%, 2 mL for 38%, and 3 mL for 45%, v/w in homogenization buffer) and ultracentrifuged in a swinging-bucket rotor SW 28 (Beckman) at 77,000*g* for 16 hours at 4°C. Around 1 mL samples were collected from each the following fractions: F1, 27%; F2, 27%/32%; F3, 32%/38%; and F4, 38%/45%. Recovered fractions were diluted 4***×*** in homogenization buffer and ultracentrifuged again (Beckman, Ti 50.2) at 120,000*g* for 2 hours at 4°C. The microsome pellets obtained from each fraction were resuspended in 60 mL of homogenization buffer. Resuspended fractions were determined by BCA for protein concentration and then added with sample buffer, aliquoted, and frozen in a –20°C freezer for later Western blot analysis. The yield of total amount of protein recovered from each fraction F1, F2, F3, and F4 was 0–0.006, 0.012–0.024, 0.03–0.06, 0.18–0.24 mg per heart, respectively.

### Western blot analysis.

Protein lysates were prepared from frozen hearts homogenized in RIPA lysis buffer as previously described (6). After protein quantification using the BCA protein assay (Bio-Rad), lysates were prepared in sample buffer (Thermo Fisher Scientific) and stored in –20°C for later analysis. On the date of experimentation, frozen samples were thawed and denatured at room temperature (RT) for 30 minutes before separation on 4%–12% Bis-Tris Gels (NuPAGE, Thermo Fisher Scientific). After transferring, membranes were fixed in methanol, blocked at RT for 1 hour with 5% BSA in 1***×*** TNT buffer, incubated overnight at 4°C with primary antibody including rabbit (rb) anti-GAPDH (2118S, Cell Signaling Technology), rb anti-IRAP (6918S, Cell Signaling Technology), rb anti-CaV1.2 (ACC-003, Alomone Labs), mouse (ms) anti-SERCA2a (ab2861, Abcam), ms anti-RyR (ab2868, Abcam), rb anti–Caveolin 3 (ab2912, Abcam), ms anti-GLUT4 (4670-1725GA, Bio-Rad Laboratories), and rb anti-BIN13 (A#5299, custom made from Anaspec and a gift from Sarcotein Diagnostics; ref. 5). After primary antibody incubation, membranes were washed and incubated with HRP or fluorescent (Alexa Fluor 555 or 647) conjugated secondary antibodies (goat [gt] anti–ms or rb IgG; A21429, A21236, 31460, 31430; Thermo Fisher Scientific) for 1 hour at RT. Immunoreactive bands were imaged with the FluorChem M imager (ProteinSimple), and band intensities were quantified with ImageJ software (NIH).

### qPCR analysis.

Total RNA was extracted from left ventricular myocardium using PureLink RNA extraction kit (Invitrogen); then, cDNA was synthesized using SuperScript IV VILO Master Mix kit (Invitrogen). We designed a custom V5 TaqMan probe to detect V5-labeled exogenous cBin1 expression (normalized to housekeeping gene Hprt1) in multiple organs collected from posttreatment mice. A custom-designed porcine cBIN1 TaqMan probe was used to detect endogenous cBIN1 in porcine hearts. V5/cBIN1 (porcine) was used to calculate the expression of exogenous cBIN1-V5 as a percentage of endogenous porcine cBIN1. TaqMan Universal PCR Master Mix was used for qPCR examination.

### Immunofluorescence labeling, spinning disc confocal microscopy, and imaging analysis.

For myocardial tissue staining, fresh heart cross sections were embedded in 100% OCT media, flash frozen in dry ice with ethanol, and stored in a –80°C freezer before being sectioned at 10 μm as previously reported (10). After acetone fixation, tissue cryosections were permeabilized with 0.1% Triton X-100 (in 5% NGS and 1***×*** PBS) for 1 hour at RT. Freshly isolated live cardiomyocytes were fixed with –20°C methanol for 5 minutes and permeabilized with 0.5% Triton X-100 (in 5% NGS and 1***×*** PBS) for 1 hour at RT. For V5, SERCA2a, GLUT4, and IRAP labeling, permeabilized and blocked tissue sections or fixed cardiomyocytes were incubated with primary antibodies against V5 (V8317-2MC, Sigma-Aldrich), SERCA2a (ab2861, Abcam), GLUT4 (4670-1725GA, Bio-Rad Laboratories), or IRAP (6918S, Cell Signaling Technology) overnight at 4°C. After washes, fluorescent (Alexa Fluor 488, 555 or 647) conjugated secondary antibodies were used to label primary antibody detection before mounting with DAPI containing Prolong Gold medium. All imaging was obtained with a Nikon Eclipse Ti microscope with a 100 × 1.49 numerical aperture total internal reflection fluorescence objective and NIS Elements software (Nikon). Confocal *Z* stacks at *Z*-step increments of 0.5 μm were collected with a SoRa spinning-disk confocal unit (Yokogawa) connected to the same Ti microscope with diode-pumped solid-state lasers (wavelength at 405, 486, 561, 647 nm) generated from laser merge module 5 (Spectral Applied Research) and captured by a high-resolution SBI digital CMOS camera.

Using the obtained confocal images, SERCA2a, GLUT4, and IRAP fluorescent intensity profiles were generated by ImageJ as previously reported (8). For power spectrum analysis, the frequency domain power spectrum of cardiomyocyte image subsections was generated in MatLab using fast Fourier transform (FFT) conversion (8, 55). Normalized t-tubule peak power density (near 1.8–2 mm) (56) was quantified and compared among groups.

### MS-based label-free quantitative (LFQ) analysis.

As described previously (57), LC-MS/MS–based LFQ was used to examine global protein abundance from heart lysates obtained from db/m + GFP, db/db + GFP, and db/db + cBIN1 groups (*n* = 4, 4, 3 hearts for each group, respectively, with 2 technical repeats per heart). Data analysis was performed using MaxQuant (v1.6.7.0) interfaced with the Andromeda search engine and subsequent analysis was done in Perseus (1.6.5.0) to generate 2-tailed *t* tests. All proteins were identified by at least one unique peptide present in at least 70% of the replicates for each group with a *P* value of less than 0.1 by 2-tailed Student’s *t* test, and a fold change > 1.25 fold between db/db + GFP and db/db + cBIN1 groups were included in the generation of the PCA plot by MetaboAnalyst 5.0 (58). For the analysis of individual proteins, quantitative data for unique peptides was extracted from this data set for SERCA2a (Atp2a2), RyR2 (Ryr2), GLUT4 (Slc2a4), and IRAP (Lnpep), and data were plotted and compared by 1-way ANOVA followed by Bonferroni’s test or Kruskal-Wallis test followed by Dunn’s test for comparison between the selected pairs.

### iGTTs and iITTs.

After fasting for 16 hours for GTTs or 4 hours for ITTs, basal blood glucose levels were measured via tail bleeding using a glucometer. A minimum of 3 days separated the glucose and ITTs. Mice were administered glucose (1 g/kg, i.p.) or insulin (0.75 IU/kg, i.p.) to assess glucose or insulin tolerance, respectively. Tail blood glucose levels (mg/dL) were subsequently measured at the times indicated.

### Statistics.

Data were analyzed using Prism 9 software (GraphPad). All quantitative data were expressed as mean ± SEM. Normality was assessed by Shapiro-Wilk test. For comparison between 2 groups, unpaired 2-tailed student’s *t* test or nonparametric Mann-Whitney *U* test was performed. For comparison among 3 groups, 1-way ANOVA followed by Bonferroni’s test or nonparametric Kruskal-Wallis test followed by Dunn’s test for selected-pair comparison were performed. For assays with multiple insulin concentrations or time points within each group, 2-way ANOVA followed by Tukey’s or Bonferroni’s test for multiple comparisons was used to determine differences among AAV9 groups. Categorical variables were analyzed using χ^2^ tests. *P* values less than 0.05 were considered statistically significant.

### Study approval.

All experimental procedures were approved by the IACUC of University of Utah and performed in accordance with NIH guidelines.

### Data availability.

The MS raw files have been uploaded to the PRIDE database (accession no. PXD044236) via the PRIDE partner repository and are publicly available. Other data that support the findings of this study are available in the Supporting Data Values file.

## Author contributions

JL, RMS, and TH conceived and designed the study. JL designed and carried out the whole-animal and isolated cardiomyocyte experiments with assistance from BR, AAC, RB, MAW, and KS and with input from SF, JR, KF, and TH. JL and AAC generated the glucose tracing data with guidance from JR. RB and SF generated the MS data. TH and JL designed and carried out the treadmill experiments with input from JDS. All authors analyzed the data. JL and TH drafted the manuscript. RMS edited the manuscript and finalized it with input from all other authors.

## Supplementary Material

Supplemental data

Supplemental data set 1

Supporting data values

## Version 1 08/28/2023

In-Press Preview

## Version 2 09/22/2023

Electronic publication
