# Supplementary material for: Cardiac gene therapy treats diabetic cardiomyopathy and lowers blood glucose
Source: JCI Insight. 2023 Sep 22;8(18):e166713. doi: 10.1172/jci.insight.166713 (PMC10561727; doi:10.1172/jci.insight.166713)
Supplement: Supplemental data [file jciinsight-8-166713-s183.pdf]

SUPPLEMENTAL MATERIAL

SUPLLEMENTAL TABLES AND DATASET

Supplemental Table 1. Echocardiography parameters in 9-weeks old *db/m* and *db/db* mice.

	db/m (n = 24-26)	*db/db* (n = 33-35)
**E** (mm/s)	496.8 ± 21.26	465.9 ± 9.98
**A** (mm/s)	316.6 ± 18.14	346.6 ± 12.28
**e'** (mm/s)	18.12 ± 1.13	13.72 ± 0.63***
**E/A**	1.63 ± 0.06	1.38 ± 0.04***
**E/e'**	29.22 ± 1.60	37.08 ± 2.46*
**MPI**	0.51 ± 0.02	0.66 ± 0.01***
**IVRT** (ms)	16.73 ± 0.65	22.77 ± 0.46***
**EF** (%)	54.93 ± 1.59	58.12 ± 1.81
**LVEDV** (µL)	48.64 ± 2.18	39.02 ± 1.18***
**LVESV** (µL)	22.02 ± 1.29	16.66 ± 1.04**
**HR** (bpm)	412.9 ± 7.40	420.7 ± 7.35
**SV** (µL)	26.62 ± 1.38	22.35 ± 0.71*
**CO** (mL/min)	10.99 ± 0.59	9.41 ± 0.34*
**LVAWs** (mm)	1.12 ± 0.04	1.28 ± 0.03**
**LVAWd** (mm)	0.74 ± 0.03	0.79 ± 0.02
**LVPWs** (mm)	0.95 ± 0.04	1.03 ± 0.02
**LVPWd** (mm)	0.70 ± 0.02	0.71 ± 0.01
**RWT**	0.36 ± 0.01	0.38 ± 0.01
**LVM** (mm)	97.46 ± 4.69	98.01 ± 2.23
**BW** (g)	24.08 ± 0.84	43.53 ± 0.75***

E, peak velocity blood flow from left ventricular relaxation in early diastole; A, peak velocity flow in late diastole caused by atrial contraction; e', early diastolic mitral annular tissue velocity; MPI, myocardial performance index; IVRT, isovolumic relaxation time; EF, ejection fraction; LVEDV, left ventricular end-diastolic volume; LVESV, left ventricular end-systolic volume; HR, heart rate; SV, stroke volume; CO, cardiac output; LVAWs/LVPWs, left ventricular end-systolic anterior/posterial wall thickness; LVAWd/LVPWd, left ventricular end-diastolic anterior/posterial wall thickness; RWT, relative wall thickness; LVM, left ventricular mass; BW, body weight. Data are presented as mean ± SEM. Unpaired Student's t-test or Mann-Whitney test is used for comparison between *db/m* and *db/db*. *, **, *** p <0.05, 0.01, or 0.001 when compared between the two groups.

Supplemental Table 2. Echocardiography parameters in 17-weeks old *db/m* and *db/db* mice at 8-weeks post PBS or AAV9 injection.

	db/m			*db/db*		
	PBS (n = 5)	AAV9-GFP (n = 11-12)	AAV9-cBIN1 (n = 9-10)	PBS (n = 5)	AAV9-GFP (n = 16)	AAV9-cBIN1 (n = 16-17)
**E** (mm/s)	620.49 ± 53.93	552.19 ± 31.29	577.25 ± 15.85	377.02 ± 25.45***	413.11 ± 20.74***	541.97 ± 13.63$^{\#\#\#, \dagger\dagger\dagger}$
**A** (mm/s)	372.36 ± 38.42	340.54 ± 30.11	359.42 ± 18.92	330.34 ± 36.15	381.86 ± 22.43	324.79 ± 18.00
**e'** (mm/s)	17.05 ± 0.91	19.32 ± 1.18	18.99 ± 1.38	10.04 ± 0.34*	12.62 ± 0.68***	21.39 ± 1.43$^{\#\#\#, \dagger\dagger\dagger}$
**E/A**	1.69 ± 0.07	1.67 ± 0.07	1.63 ± 0.07	1.19 ± 0.13*	1.09 ± 0.03***	1.75 ± 0.10$^{\#\#\#, \dagger\dagger\dagger}$
**E/e'**	36.24 ± 1.94	30.12 ± 2.97	31.75 ± 2.47	37.55 ± 2.13	33.64 ± 1.95	26.59 ± 1.28$^{\#, \dagger}$
**MPI**	0.44 ± 0.04	0.54 ± 0.03	0.51 ± 0.02	0.89 ± 0.05***	0.78 ± 0.03***	0.48 ± 0.02$^{\#\#\#, \dagger\dagger\dagger}$
**IVRT** (ms)	13.78 ± 1.67	16.55 ± 1.24	17.32 ± 1.06	29.86 ± 1.32***	24.50 ± 0.90***$^{, \#\#}$	15.22 ± 0.53$^{\#\#\#, \dagger\dagger\dagger}$
**EF** (%)	51.71 ± 2.72	52.79 ± 3.11	60.91 ± 2.98†	64.30 ± 2.15*	62.16 ± 1.74**	66.67 ± 1.43
**LVEDV** (µL)	68.91 ± 2.88	61.85 ± 4.38	53.56 ± 4.17$^{\#}$	39.28 ± 1.43***	36.77 ± 1.53***	52.27 ± 2.80†††
**LVESV** (µL)	33.49 ± 2.81	29.75 ± 3.34	21.77 ± 3.20$^{\#, \dagger}$	14.13 ± 1.30***	14.14 ± 1.05***	17.84 ± 1.49
**HR** (bpm)	397.25 ± 17.94	438.26 ± 7.48	424.10 ± 19.88	410.16 ± 13.12	443.54 ± 9.57	444.31 ± 11.37
**SV** (µL)	35.42 ± 1.41	32.10 ± 2.57	31.79 ± 1.47	25.15 ± 0.53*	22.63 ± 0.80***	34.43 ± 1.51$^{\#\#, \dagger\dagger\dagger}$
**CO** (mL/min)	14.11 ± 0.95	15.03 ± 1.82	13.50 ± 0.93	10.32 ± 0.37	10.00 ± 0.34***	15.06 ± 0.68$^{\#, \dagger\dagger\dagger}$
**LVAWs** (mm)	1.37 ± 0.07	1.22 ± 0.05	1.21 ± 0.03	1.77 ± 0.06**	1.36 ± 0.06$^{\#\#\#}$	1.46 ± 0.04**$^{, \#\#}$
**LVAWd** (mm)	0.88 ± 0.09	0.79 ± 0.05	0.81 ± 0.03	0.96 ± 0.05	0.90 ± 0.04*	0.87 ± 0.02
**LVPWs** (mm)	1.05 ± 0.06	1.03 ± 0.05	1.08 ± 0.07	1.56 ± 0.13***	1.25 ± 0.05*$^{, \#}$	1.32 ± 0.05*
**LVPWd** (mm)	0.78 ± 0.03	0.76 ± 0.05	0.76 ± 0.06	0.95 ± 0.07	0.84 ± 0.03	0.87 ± 0.04
**RWT**	0.35 ± 0.02	0.37 ± 0.02	0.38 ± 0.03	0.59 ± 0.06***	0.47 ± 0.01**$^{, \#\#}$	0.46 ± 0.02*$^{, \#\#}$
**LVM** (mm)	147.38 ± 8.65	120.87 ± 8.78$^{\#}$	117.17 ± 8.58$^{\#}$	110.44 ± 5.47*	112.58 ± 6.09	122.63 ± 5.51
**BW** (g)	28.30 ± 0.65	27.25 ± 1.06	26.89 ± 0.62	50.12 ± 2.20***	52.50 ± 0.87***	52.85 ± 0.87***

E, peak velocity blood flow from left ventricular relaxation in early diastole; A, peak velocity flow in late diastole caused by atrial contraction; e', early diastolic mitral annular tissue velocity; MPI, myocardial performance index; IVRT, isovolumic relaxation time; EF, ejection fraction; LVEDV, left ventricular end-diastolic volume; LVESV, left ventricular end-systolic volume; HR, heart rate; SV, stroke volume; CO, cardiac output; LVAWs/LVPWs, left ventricular end-systolic anterior/posterior wall thickness; LVAWd/LVPWd, left ventricular end-diastolic anterior/posterior wall thickness; RWT, relative wall thickness; LVM, left ventricular mass; BW, body weight. Data are presented as mean ± SEM. Two-way ANOVA followed by Tucky's or Bonferroni's test is used for comparison among groups. *, **, *** p <0.05, 0.01, or 0.001 when compared between two different genotype groups within the same AAV9-treatment. $^{\#}$, $^{\#\#}$, $^{\#\#\#}$ p <0.05, 0.01, or 0.001 when compared to PBS group within the same genotype. †, ††, ††† p <0.05, 0.01, or 0.001 when compared to AAV9-GFP group within the same genotype.

Supplemental Table 3. Echocardiography parameters and treadmill performance in 8-months old *db/m* and *db/db* mice at 8-weeks post AAV9-CMV-GFP/cBIN1 injection.

	db/m-GFP (n = 8)	*db/db*-GFP (n = 7)	*db/db*-cBIN1 (n = 8)
**E** (mm/s)	576.0 ± 49.09	504.8 ± 44.30	536.2 ± 38.44
**A** (mm/s)	351.3 ± 32.92	356.5 ± 20.37	344.8 ± 19.18
**e'** (mm/s)	18.32 ± 1.94	14.81 ± 2.06	20.97 ± 1.07†
**E/A**	1.67 ± 0.09	1.44 ± 0.14	1.57 ± 0.11
**E/e'**	33.11 ± 3.76	36.93 ± 4.74	25.80 ± 1.82†
**MPI**	0.55 ± 0.03	0.71 ± 0.03*	0.56 ± 0.04†
**IVRT** (ms)	21.72 ± 0.81	28.15 ± 1.02***	21.48 ± 0.62†††
**EF** (%)	70.93 ± 3.49	74.20 ± 2.04	75.63 ± 1.68
**LVEDV** (µL)	50.10 ± 3.29	38.86 ± 2.40*	46.77 ± 3.25
**LVESV** (µL)	14.46 ± 1.81	10.25 ± 1.28	11.27 ± 1.46
**HR** (bpm)	437.0 ± 17.61	425.4 ± 26.76	420.3 ± 10.37
**SV** (µL)	35.65 ± 2.65	28.61 ± 1.27*	35.51 ± 2.27†
**CO** (mL/min)	15.44 ± 1.02	12.18 ± 0.97*	14.80 ± 1.13
**LVAWs** (mm)	1.81 ± 0.06	1.88 ± 0.05	1.78 ± 0.04
**LVAWd** (mm)	1.18 ± 0.06	1.11 ± 0.07	1.07 ± 0.04
**LVPWs** (mm)	1.35 ± 0.05	1.32 ± 0.05	1.34 ± 0.09
**LVPWd** (mm)	0.78 ± 0.03	0.80 ± 0.04	0.78 ± 0.03
**RWT**	0.48 ± 0.03	0.45 ± 0.03	0.45 ± 0.02
**LVM** (mm)	118.8 ± 11.32	126.4 ± 5.72	114.8 ± 4.38
**BW** (g)	29.66 ± 0.86	40.94 ± 1.70***	40.76 ± 2.17***
**Maximal Distance** (m)	191.1 ± 3.92	25.49 ± 4.56***	49.03 ± 7.24***, ††

E, peak velocity blood flow from left ventricular relaxation in early diastole; A, peak velocity flow in late diastole caused by atrial contraction; MPI, myocardial performance index; IVRT, isovolumic relaxation time; EF, ejection fraction; LVEDV, left ventricular end-diastolic volume; LVESV, left ventricular end-systolic volume; HR, heart rate; SV, stroke volume; CO, cardiac output; LVAWs, left ventricular end-systolic anterior wall thickness; LVAWd, left ventricular end-diastolic anterior wall thickness; LVPWs, left ventricular end-systolic posterior wall thickness; LVPWd, left ventricular end-diastolic posterior wall thickness; RWT, relative wall thickness; LVM, left ventricular mass; BW, body weight; HW/TL, heart weight/ tibial length; LW/TL, lung weight/ tibial length. Data are presented as mean ± SEM. One-way ANOVA followed by Bonferroni's test or Kruskal-Wallis test followed by Dunn's test for selected pair comparison was performed. *, **, *** p <0.05, 0.01, or 0.001 for comparison vs. *db/m*-GFP; †, ††, ††† p <0.05, 0.01, or 0.001 for comparison vs. *db/db*-GFP.

Supplemental Table 4. Echocardiography parameters and treadmill performance in 17-weeks old *db/m* and *db/db* mice at 8-weeks post AAV9-cTnT-GFP/cBIN1 injection.

	db/m-GFP (n = 12)	*db/db*-GFP (n = 12)	*db/db*-cBIN1 (n = 12)
**E** (mm/s)	559.2 ± 36.55	397.7 ± 28.52***	536.1 ± 25.24††
**A** (mm/s)	337.5 ± 29.06	351.9 ± 24.05	355.6 ± 21.06
**e'** (mm/s)	18.11 ± 1.38	9.97 ± 0.87***	19.48 ± 0.86†††
**E/A**	1.70 ± 0.07	1.14 ± 0.05***	1.53 ± 0.06††
**E/e'**	32.56 ±2.74	42.88 ± 5.07	28.11 ± 1.87††
**MPI**	0.49 ± 0.02	0.79 ± 0.04***	0.49 ± 0.01†††
**IVRT** (ms)	15.20 ± 0.66	26.70 ± 1.47***	16.15 ± 0.34†††
**EF** (%)	74.68 ± 2.33	74.65 ± 2.80	78.32 ± 1.82
**LVEDV** (μL)	49.31 ± 3.03	35.50 ± 2.42***	46.29 ± 1.99††
**LVESV** (μL)	13.03 ± 1.74	9.38 ± 1.55	10.16 ± 1.00
**HR** (bpm)	403.5 ± 10.60	394.6 ± 14.77	421.6 ± 11.82
**SV** (μL)	36.28 ± 1.73	26.12 ± 1.68***	36.13 ± 1.56†††
**CO** (mL/min)	16.50 ± 0.69	10.42 ± 0.56***	15.45 ± 0.65†††
**LVAWs** (mm)	1.58 ± 0.06	1.56 ± 0.07	1.70 ± 0.06
**LVAWd** (mm)	0.96 ± 0.04	0.95 ± 0.05	0.98 ± 0.06
**LVPWs** (mm)	1.29 ± 0.08	1.27 ± 0.05	1.36 ± 0.06
**LVPWd** (mm)	0.87 ± 0.05	0.83 ± 0.03	0.87 ± 0.03
**RWT**	0.50 ± 0.03	0.51 ± 0.04	0.47 ± 0.02
**LVM** (mm)	116.7 ± 5.20	103.3 ± 4.46	128.9 ± 5.30†††
**BW** (g)	27.51 ± 1.31	49.18 ± 0.90***	48.11 ± 0.99***
**Maximal Distance** (m)	208.8 ± 4.80	44.26 ± 4.63***	80.98 ± 7.96***, †††

E, peak velocity blood flow from left ventricular relaxation in early diastole; A, peak velocity flow in late diastole caused by atrial contraction; MPI, myocardial performance index; IVRT, isovolumic relaxation time; EF, ejection fraction; LVEDV, left ventricular end-diastolic volume; LVESV, left ventricular end-systolic volume; HR, heart rate; SV, stroke volume; CO, cardiac output; LVAWs, left ventricular end-systolic anterior wall thickness; LVAWd, left ventricular end-diastolic anterior wall thickness; LVPWs, left ventricular end-systolic posterior wall thickness; LVPWd, left ventricular end-diastolic posterior wall thickness; RWT, relative wall thickness; LVM, left ventricular mass; BW, body weight; HW/TL, heart weight/ tibial length; LW/TL, lung weight/ tibial length. Data are presented as mean ± SEM. One-way ANOVA followed by Bonferroni's test or Kruskal-Wallis test followed by Dunn's test is used for selected pair comparison. *, **, *** p <0.05, 0.01, or 0.001 for comparison vs. *db/m*-GFP; †, ††, ††† p <0.05, 0.01, or 0.001 for comparison vs. *db/db*-GFP.

Supplemental Dataset 1. All 1212 proteins detected by mass spectrometry and their comparisons between GFP and cBIN1 treatment in *db/db* mice.

- **Please see the attached .xls file for the dataset.**

SUPPLEMENTAL FIGURES AND FIGURE LEGENDS

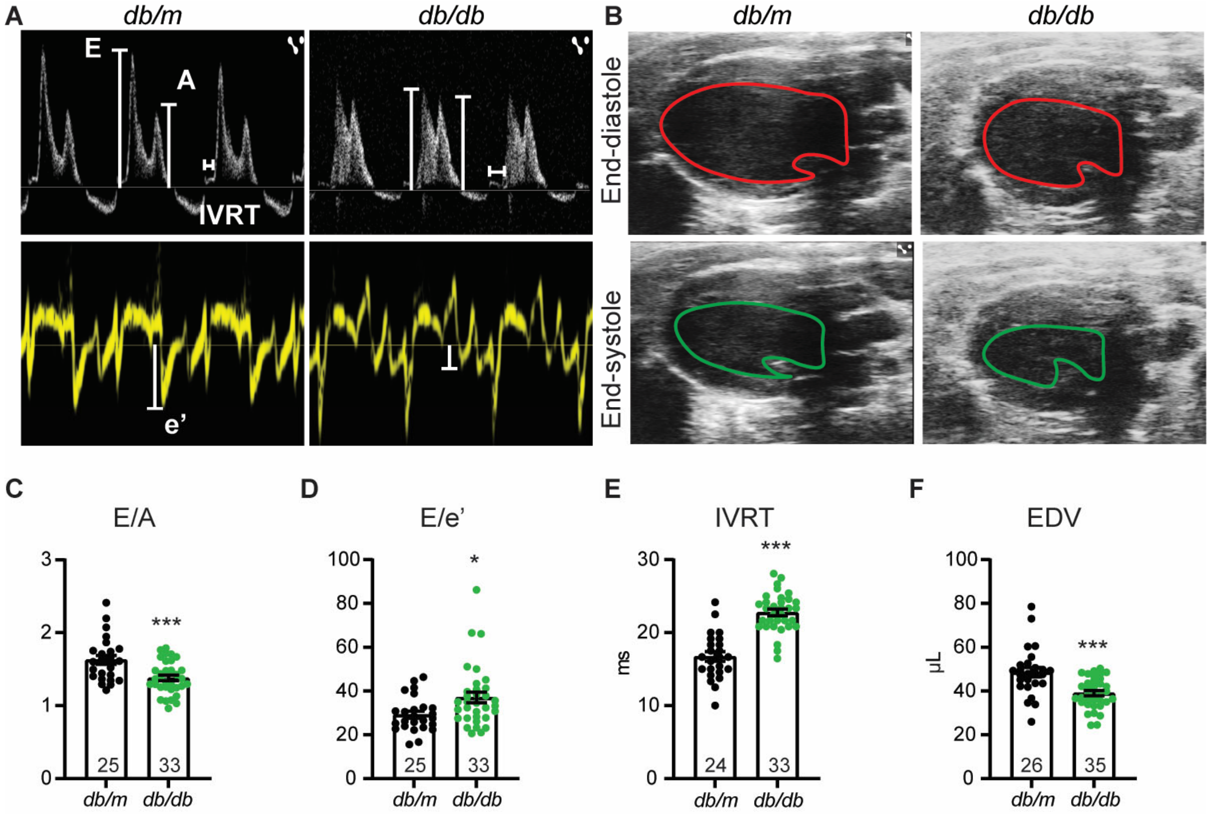

Supplemental Figure 1. ***db/db*** **mice develop diastolic dysfunction at 9 weeks of age.** (**A**) Representative mitral valve inflow pulsed wave Doppler images (top) and tissue Doppler images of septal mitral valve annulus (bottom), and (**B**) longitudinal axis view of left ventricles at end diastolic (top) and end systolic (bottom) phase in 9-weeks old *db/m* and *db/db* mice. (**C**-**F**) Quantitative analysis of E/A (**C**), E/e' (**D**), isovolumic relaxation time (IVRT) (**E**), and end-diastolic volume (EDV) (**F**) (n = 24-35). All data are presented as mean ± SEM. Unpaired Student's t-test is used for comparison between *db/m* and *db/db*. *, *** indicates p<0.05, 0.001 for comparison vs. *db/m*.

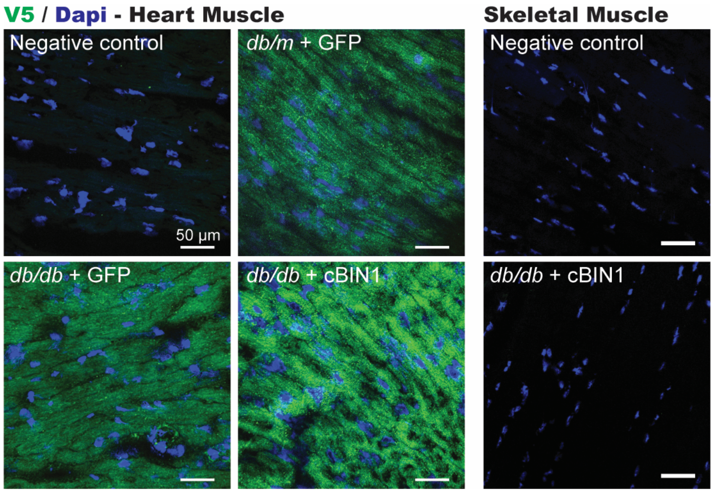

Supplemental Figure 2. Fluorescent confocal imaging confirms exogenous protein transduction in heart but not skeletal muscle in mice after AAV9 injection. Representative immunostaining images of myocardial tissue stained with rabbit anti-V5 antibody. V5-tagged exogenous protein was transduced into nearly 100% cardiomyocytes by AAV9 virus (left two panels) but not skeletal muscle cells (right panel). The negative control from mice without AAV9 injection was treated with the same immunofluorescent labeling procedure including primary and secondary antibodies. Scale bar, 50 μm.

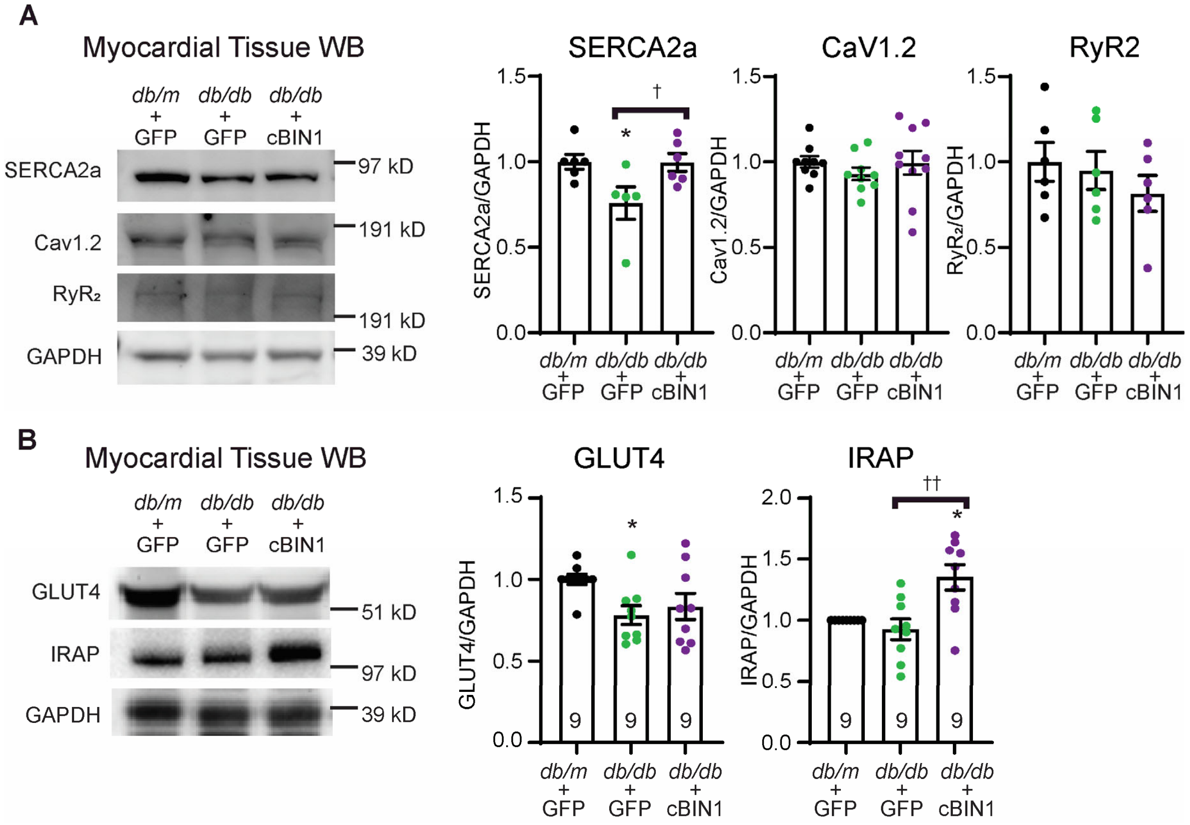

Supplemental Figure 3. Protein expression of calcium handling and GLUT4 translocation proteins in AAV9-treated mice. (**A**-**B**) Representative Western blots of total RyR2, CaV1.2, SERCA2a (**A**) and GLUT4, IRAP (**B**) protein expressions in myocardial tissue from *db/m* + GFP, *db/db* + GFP, and *db/db* + cBIN1 hearts at 17 weeks with quantifications to the right (n = 5-10 hearts per group). All data are presented as mean ± SEM. One-way ANOVA followed by Bonferroni's test or Kruskal-Wallis test followed by Dunn's test is used for selected pair comparison. * indicates p<0.05 for comparison vs. *db/m* + GFP; †, †† indicates p<0.05, 0.01 for comparison between *db/db* +GFP and *db/db* + cBIN1.

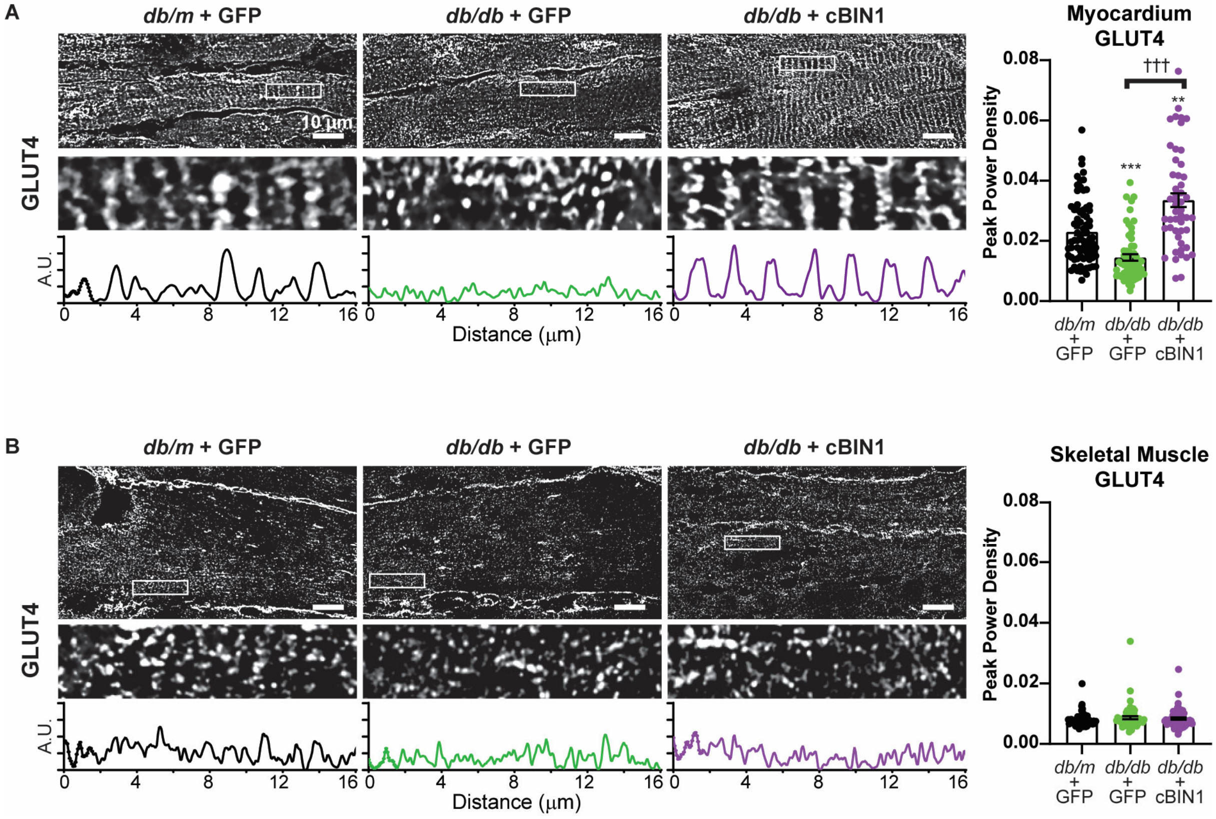

Supplemental Figure 4. Intracellular GLUT4 distribution in myocardial and skeletal muscle cryo-sections. Representative GLUT4 confocal images in myocardial (n = 50-74 cells from 3 hearts, **A**) and skeletal muscle (n = 62-66 cells from 3 mice, **B**) cryo-sections with corresponding fluorescence intensity profiles of the boxed areas (bottom panels) and quantification of GLUT4 peak power density (right panel) at t-tubules (scale bar, 10 µm). Data are presented as mean ± SEM. Kruskal-Wallis test followed by Dunn's test is used for selected pair comparison. *, **, *** indicates $p<0.05$, 0.01, and 0.001 for comparison vs. *db/m* + GFP; †, ††, ††† indicates $p<0.05$, 0.01, 0.001 for comparison between *db/db* + GFP and *db/db* + cBIN1.
